# Exploring the therapeutic potential of marjoram (*Origanum majorana* L.) in polycystic ovary syndrome: insights from serum metabolomics, network pharmacology and experimental validation

**DOI:** 10.1186/s12906-025-04774-5

**Published:** 2025-02-21

**Authors:** Aliaa M. Elfiky, Reham S. Ibrahim, Amira R. Khattab, Mai O. Kadry, Naglaa M. Ammar, Eman Shawky

**Affiliations:** 1 Central Administration of Pharmaceutical Care, General Administration of Pharmaceutical Vigilance, Egyptian Drug Authority, Cairo, Egypt; 2 Department of Pharmacognosy, Faculty of Pharmacy, Alexandria University, Alexandria, 21521 Egypt; 3 Pharmacognosy Department, College of Pharmacy, Arab Academy for Science, Technology and Maritime Transport, Alexandria, 1029 Egypt; 4 Therapeutic Chemistry Department, National Research Center, Al Bouhouth Street, Dokki, Giza, 12066 Egypt

**Keywords:** Polycystic ovary syndrome, Network pharmacology, Marjoram, UPLC-MS/MS, Serum pharmacochemistry

## Abstract

**Background:**

Polycystic Ovary Syndrome (PCOS) is a common endocrine disorder with significant metabolic and hormonal dysregulation. Marjoram (*Origanum majorana* L.), known for its medicinal properties, has potential in managing PCOS through various bioactive compounds.

**Objective:**

This study aims to evaluate the effects of marjoram on PCOS symptoms using serum pharmacochemistry, network pharmacology, and molecular docking in a DHEA-induced rat model.

**Methods:**

Polycystic Ovary Syndrome (PCOS) was induced in rats using dehydroepiandrosterone (DHEA). Marjoram’s therapeutic effects were evaluated by analyzing oxidative stress biomarkers, hormone levels, and ovarian histopathology. Untargeted serum metabolomics, conducted with ultra-high-performance liquid chromatography-tandem mass spectrometry (UHPLC TQD-MS/MS), identified key bioactive compounds. These compounds were then examined through network pharmacology to map their interactions with PCOS-related pathways, with findings validated via molecular docking.

**Results:**

Marjoram treatment significantly reduced oxidative stress by decreasing nitric oxide (NO) and increasing total antioxidant capacity (TAC). Hormonal analysis revealed that high-dose marjoram (100 mg/kg) normalized progesterone, estradiol, testosterone and FSH levels. Body weight gain was also reduced with marjoram treatment, especially at the higher dose. Histopathological evaluation showed fewer ovarian cysts and improved follicular structure with marjoram administration. Network pharmacology analysis highlighted the steroid hormone biosynthesis and estrogen signaling pathways as critical targets, with apigenin and oleic acid identified as active compounds. Molecular docking confirmed strong interactions of these compounds with core PCOS-associated proteins, further supporting marjoram’s potential in modulating PCOS symptoms.

**Conclusion:**

This study reveals that marjoram contains a diverse range of active compounds that can modulate crucial biochemical and histological markers related to PCOS. By combining serum pharmacochemistry with network pharmacology, the research highlights marjoram’s potential as a natural supplement to help alleviate PCOS symptoms and slow the syndrome’s progression. These findings support further investigation into marjoram’s role as a complementary therapy for managing PCOS.

**Supplementary Information:**

The online version contains supplementary material available at 10.1186/s12906-025-04774-5.

## Introduction

Polycystic ovary syndrome (PCOS) is a prevalent endocrine disorder affecting 6–20% of women of reproductive age and is characterized by genetic, metabolic, and hormonal complexities. Common symptoms include irregular menstrual cycles, hyperandrogenism, insulin resistance, and chronic inflammation, contributing to metabolic and reproductive complications [1]. Classified as an inflammatory and possibly autoimmune condition, PCOS affects multiple organ systems, making treatment challenging. Current therapies include hormonal treatments, ovulation induction, and oral contraceptives, tailored based on symptoms such as androgen excess, menstrual irregularities, and fertility goals. However, these conventional therapies face significant limitations, including adverse effects, poor patient adherence, and unsatisfactory outcomes, emphasizing the urgent need for safer and more effective treatment options [2].

Advances in understanding PCOS pathophysiology underscore the significant roles of chronic inflammation, oxidative stress, and insulin resistance. Cytokines, growth factors, and reactive oxygen species released by the ovaries, liver, and other tissues contribute to systemic inflammation and metabolic imbalances. Oxidative stress, a key factor, results from reduced endogenous antioxidants, leading to oxidative damage in ovarian tissues and worsening the condition [3]. Studies demonstrate that antioxidant supplementation can enhance enzyme activity, such as superoxide dismutase (SOD) and glutathione peroxidase (GPx), while reducing oxidative stress biomarkers, including malondialdehyde (MDA) and lipid peroxides, thereby restoring the antioxidant-oxidant balance in PCOS patients [4].

Insulin resistance and hyperinsulinemia are central to PCOS pathophysiology, profoundly impacting the hypothalamic-pituitary-ovarian (HPO) axis. These metabolic disruptions lead to anovulation, hirsutism, obesity, and ovarian cysts. Elevated luteinizing hormone (LH) levels, driven by increased gonadotropin-releasing hormone (GnRH) pulse frequency and amplitude, play a critical role in PCOS. Abnormal LH stimulation exacerbates ovarian dysfunction and androgen production, sustaining the syndrome. Treatments targeting GnRH pulse frequency, such as GnRH agonists, have been shown to reduce LH and androgen levels, providing insights into hormonal management strategies [5].

Pharmaceutical treatments like clomiphene and metformin are widely used but have limitations due to adverse reactions, compliance issues, and limited therapeutic efficacy [6]. To address these gaps, preclinical studies frequently employ dehydroepiandrosterone (DHEA)-induced animal models to mimic key PCOS features, such as polycystic ovaries and anovulation. Although these models do not fully replicate human PCOS, they are valuable for testing therapies that target PCOS morphology and hormonal imbalances without inducing systemic metabolic abnormalities [7].

Marjoram (*Origanum majorana* L.), a traditional herb native to Egypt and the Eastern Mediterranean, has emerged as a promising natural remedy for PCOS. Traditionally used to treat hormonal imbalances, early studies suggest that marjoram improves insulin sensitivity, reduces DHEA-S and fasting insulin levels, and modulates inflammatory markers and oxidative stress [8, 9]. Recent research demonstrates that marjoram increases estradiol levels, decreases insulin and glucose concentrations, and addresses hormonal imbalances, making it a unique herbal remedy with ovulation-inducing effects [10]. These effects are supported by clinical trials, highlighting marjoram’s potential as a natural alternative for ovulation induction in women with PCOS.

Marjoram’s bioactive components, including flavonoids and phenolic compounds, exhibit potent antioxidant activity, countering oxidative stress commonly associated with PCOS. Mechanistically, marjoram activates peroxisome proliferator-activated receptors (PPARs), enhancing insulin sensitivity and reducing metabolic dysfunctions. Studies have further highlighted its antihyperlipidemic and antidiabetic properties, particularly in DHEA-induced PCOS models, showing reductions in glucose, testosterone, and LDL-C levels while increasing HDL-C and estradiol [11]. These benefits extend to cardiovascular health, addressing a critical aspect of PCOS-related complications.

This study comprehensively investigates marjoram’s (*Origanum majorana* L.) therapeutic potential for managing Polycystic Ovary Syndrome (PCOS) through serum metabolomics (UHPLC TQD-MS/MS), network pharmacology, and molecular docking. It explores bioactive compounds like apigenin and oleic acid, identifying their interactions with metabolic and hormonal pathways. Building on prior findings of marjoram’s benefits in improving insulin sensitivity and reducing oxidative stress, the research further clarifies compound-target-pathway mechanisms.

Using a DHEA-induced rat model, the study assesses marjoram’s effects on oxidative stress, hormone levels, body weight, and ovarian histology. Molecular docking validates the binding affinities of its compounds on PCOS-related pathways, demonstrating its efficacy. The findings reinforce marjoram’s role as a complementary therapy, highlighting its potential as a natural alternative to traditional PCOS treatments with limited effectiveness.

## Results and discussion

### Biochemical characteristics

#### Oxidative stress modulation

DHEA induced oxidative stress, as shown by a significant increase in nitric oxide (NO) levels and a decrease in total antioxidant capacity (TAC). These effects were modulated following marjoram treatment, with the high dose (100 mg/kg) of marjoram showing the most pronounced impact. DHEA elevated NO and reduced TAC with a mean value of 74.07 ± 3.4 μmol/L & 5.20 ± 0.6 mmol/L, respectively. Meanwhile, marjoram high dose reduced NO and elevated TAC with a mean value of 25.90 ± 1.8 μmol/L & 7.26 ± 2.3 mmol/L, respectively as represented in Table 1.

**Table 1:** Effect of marjoram administration on oxidative biomarkers, hormonal levels and body weight gain in all experimental rat groups

Groups/parameter	Control	DHEA	marjoram (50 mg/kg b.wt)	marjoram (100 mg/ kg b.wt)
NO μmol/L	25.90 ± 2.10^a^	74.07 ± 3.40^b^	35.10 ± 2.90^c^	25.90 ± 1.80^a^
TAC mmol/L	7.29 ± 0.90^a^	5.20 ± 0.60^b^	7.00 ± 1.10^a^	7.26 ± 2.30^a^
Progesterone (ng/ml)	19.80 ± 3.10^a^	1.30 ± 0.50^b^	8.86 ± 1.10^c^	13.00 ± 2.60^d^
Estradiol (pg/ml)	398.53 ± 5.20^a^	40.00 ± 3.90^b^	183.13 ± 4.80^c^	270.35 ± 2.80^d^
FSH (ng/ml)	42.86 ± 1.65^a^	7.16 ± 0.49^b^	19.33 ± 0.52^c^	30.10 ± 1.40^d^
Testosterone (ng/ml)	5.70 ± 0.56^a^	28.40 ± 1.06^b^	14.80 ± 0.34^c^	9.26 ± 0.31^d^
Body weight gain	19.00 ± 2.90^c^	53.90 ± 2.80^a^	35.96 ± 4.70^b^	28.45 ± 4.20^c^

In the current study, DHEA induced oxidative stress, as evidenced by a reduced TAC level and elevated NO level (Table 1). These effects were modulated following marjoram treatment, with the high dose (100 mg/kg) showing superior efficacy, as highlighted by significant improvements compared to the control group.

In the whole sample, correlation analysis revealed a significant negative correlation between TAC and estradiol. According to prior research, oxidative stress may have a role in the pathophysiology of PCOS. As a result, oxidative stress parameters may be recommended as diagnostic markers for the early identification of high-risk populations. Furthermore, the research offers corroborating proof that increased oxidative stress in PCOS may be a result of obesity and sex hormones, specifically progesterone and estradiol [2]. According to a meta-analysis study by Murri et al., 2013, women with PCOS had abnormal levels of circulating oxidative stress markers, such as MDA and NO [5]. This suggests that oxidative stress may play a role in the pathophysiology of PCOS by altering steroidogenesis in the ovaries, which in turn affects follicular development, increases androgen levels, and leads to infertility [6]. Nuñez-Calonge et al., 2016 reported that PCOS decreased TAC, which was directly linked to lower rates of oocyte maturation and fertilization, poor quality embryos, and fewer pregnancy occurrences. In accordance with our results Sulaiman et al., 2018 showed that TAC levels also have a tendency to be lower in PCOS cases compared to controls. The class of non-enzymatic antioxidants known as total antioxidant capacity (TAC) represents the antioxidants’ power to prevent damage to cells caused by oxidative stress (OS). Rats with PCOS had noticeably reduced serum TAC levels, which may indicate higher OS in these rats. On the other hand, total oxidant status (TOS) is much higher when the serum level of TAC is significantly lower [9, 12]. Many studies have investigated the antioxidant and free radical-scavenging properties of marjoram. Polyphenolic compounds identified in marjoram, predominantly flavonoids, have shown significant antioxidant activity. Among these, rosmarinic acid has been found to be the strongest antioxidant polyphenol in marjoram [11].

#### Hormonal disturbance modulation

DHEA administration led to a significant hormonal imbalance, characterized by a marked decrease in the levels of progesterone, estradiol and FSH on the other hand testosterone level was elevated. Following treatment with marjoram, these hormonal levels were adjusted, with the higher dose (100 mg/Kg) of marjoram demonstrating a more pronounced effect in moderating these changes. DHEA administration led to reductions in progesterone, estradiol, and FSH levels, with mean values recorded at 1.30 ± 0.5 ng/ml, 40.00 ± 3.9 pg/ml, and 7.16 ± 0.49 ng/ml, respectively. However, it raised testosterone levels to a mean of 28.4 ± 1.06 ng/ml. In contrast, a high dose of marjoram (100 mg/kg) increased progesterone, estradiol, and FSH levels, achieving mean values of 13.00 ± 2.6 ng/ml, 270.35 ± 2.8 pg/ml, and 30.1 ± 1.4 ng/ml, respectively, while testosterone levels decreased to a mean of 9.26 ± 0.31 ng/ml, as shown in Table 1.

Because progesterone and estradiol are key hormones that regulate the reproductive system and other physiological processes, measuring the levels of these hormones in rats is critical to knowing their hormonal condition and reproductive health. Herein, DHEA triggered a hormonal imbalance, marked by lowered estradiol, progesterone and FSH levels and elevated testosterone level. However, this was modified after administering marjoram, particularly with a higher dosage (100 mg/Kg) showing more effectiveness (Table 1). Such findings highlight the importance of hyperandrogenism in the diagnosis of PCOS. In the current study, we found that the PCOS-induced group had significantly different levels of these hormones than the control group. The PCOS group had considerably lower levels of progesterone, estradiol, and FSH on the other hand elevated testosterone level indicating a disturbance in the normal hormonal balance [13].

The plasma progesterone levels in the DHEA-treated rats were low in comparison to the control rats. Anovulation may be the cause of the decrease in plasma progesterone levels observed in DHEA-treated rats [14, 15]. Furthermore, serous estradiol falls and has a causal association to hyperandrogenism in PCOS patients and the animal disease model induced by DHEA, pending the formation of follicular cysts, disturbance of the estrous cycle, aberrant ovarian steroidogenesis, and anovulation [16]. Progesterone levels in the blood fall as a result of anovulation and a reduction in corpora lutea [17]. Our findings are in line with Omnia et al., 2022 finding that progesterone levels fall following PCOS induction.

The granulosa cell-produced aromatase in the ovaries transforms C-19 androgens into estradiol. Reduced activity of this enzyme may be anticipated in PCOS, which may result in elevated ovarian androgen, decreased ovarian estrogen, and the development of PCOS in animal models [18]. According to Ashraf et al., 2019 an imbalance in reproductive hormones, including an excess of androgens (testosterone), may also contribute to a drop in serum estradiol levels in PCOS. The ovaries’ normal functions, including the synthesis of estradiol, may be disrupted with by this hormonal imbalance [19].

Moreover, key hormones regulating women’s fertility, specifically folliculogenesis, are estradiol (E2) and FSH. This steroid regulates the growth and selection of prominent preovulatory follicles and is generated locally by granulosa cells (GC) within ovarian follicles. Follicle development is frequently interrupted in PCOS, resulting in improper follicular maturation. This lack of follicular growth results in decreased estradiol production because the granulosa cells of mature follicles are the primary source of estradiol [20].

As seen by increased levels of progesterone and estradiol, marjoram reduced the harmful effects of DHEA. It has a variety of effects, such as increasing the levels of progesterone and estradiol, which are essential for females to have a regular menstrual cycle and may stimulate the development of follicles and lead to ovulation. Marjoram regulates these hormone levels, which corrects hormonal imbalances linked to PCOS. Among the two different doses of marjoram, the 100 mg/kg dose showed the most beneficial effect on these hormonal modifications (Table 1). These results align with the findings of Ibrahim et al., 2023, which reported a significant decrease in estradiol and progesterone levels when marjoram was used to treat PCOS, suggesting that marjoram may influence adrenal androgen production mechanisms, where chlorogenic acid, a phenolic compound in marjoram, was found to increase progesterone production while reducing LH and testosterone levels. Additionally, gallic acid, another major phenolic content in marjoram, reverses metabolic and endocrine abnormalities associated with letrozole (LETZ)-induced PCOS by regulating androgen and adiponectin circulation [21].

Additionally, Hussein & Alzubaidi, 2022 demonstrated a significant increase in follicle-stimulating hormone (FSH), estrogen, and progesterone levels when treated with an aqueous extract of *Origanum majorana* leaves. Their research indicated that the methanolic extract of *Origanum majorana* leaves at maximum doses stimulated sex hormones, potentially affecting the activity of the pituitary-gonadal axis and increasing the secretion of reproductive hormones [22].

### Body weight gain (BWG) modulation

The Body Weight Gain (BWG) experienced a notable increase following the induction of Polycystic Ovary Syndrome (PCOS) using DHEA, with a significant percentage change of 53.9%. This increase was subsequently mitigated after the administration of marjoram treatment. Notably, the higher dosage of marjoram (100 mg/Kg) was more effective, reducing the BWG to 28.4%, as illustrated in Table 1.

Kim et al., 2018 observed that rats developed PCOS showed a similar pattern of increased body weight following DHEA injections. Furthermore, A prior study has long shown a connection between PCOS and obesity [24]. On the other hand, some research suggests that the higher obesity rate among PCOS women relative to the control group could be a result of overweight women reporting their condition [25]. Hence, a causal role of obesity in PCOS is unlikely [26]. Our experimental model reproduces several of PCOS symptoms. The weight gain that we saw in the rats given DHEA injections could be attributed to either the androgen injection’s side effects or the PCOS phenotype [27]. In our study (Table 1) the DHEA/PCOS rats treated with marjoram had lower average body weights than the PCOS rats that were not treated; this data may point to marjoram’s potential as an anti-obesity. This result is in agreement with Negm et al., 2017 as marjoram consumption resulted in significant weight reduction after one month [28].

Values are expressed as Mean ± SEM. Level of significance was detected when *P* < 0.05. DHEA: dehydroepiandrosterone. Similar letters are not considered significantly different from each other while different letters are considered significantly different.

### Histopathological investigations

**Fig. 1:**
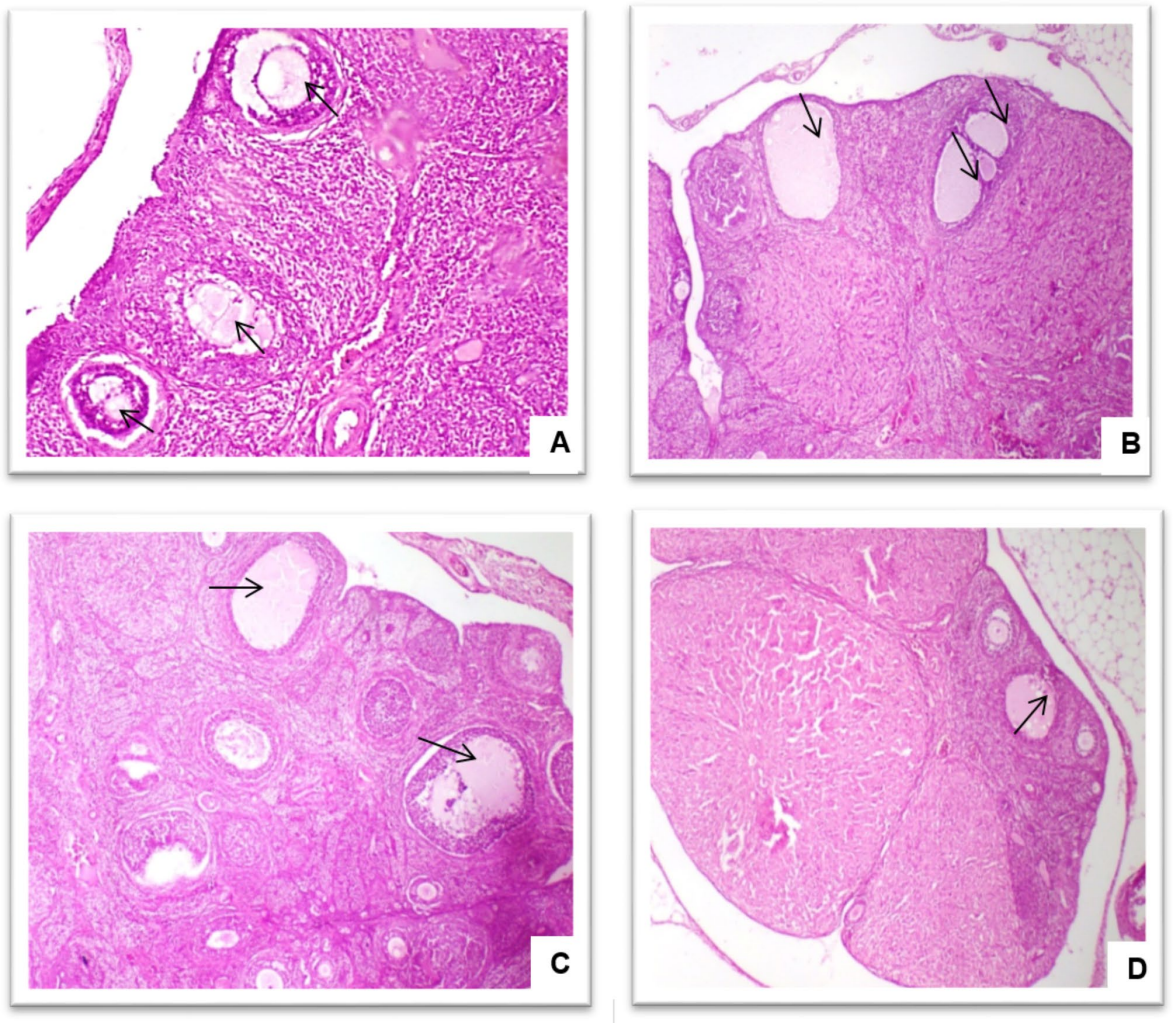
(**A**) Control group: Photomicrograph, rat ovary showing normal histological structure of ovarian follicles (arrows). (**B**) DHEA: Photomicrograph, rat ovary showing multiple ovarian follicular cysts (arrows). (**C**) marjoram Low dose (50 mg/Kg): Photomicrograph, rat ovary showing ovarian follicular cysts (arrows). (**D**) marjoram High dose (100 mg/Kg): Photomicrograph, rat ovary showing one ovarian cyst (arrow) (H&EX100)

As shown in Fig. 1, control group showed normal histological structure of ovarian follicles. PCOS group revealed presence of multiple ovarian cysts (polycystic ovaries). Marjoram (low dose) revealed presence of few ovarian follicles with moderate vacuolar degeneration while marjoram high dose (100 mg/Kg) showed amelioration in the abovementioned lesions as the ovarian cysts were fewer. Recorded lesions in ovaries were scored according to their severity. The control group showed no ovarian cysts, scoring 0. Meanwhile, the DHEA group exhibited significant lesions, scoring 3. The marjoram-treated groups showed a dose-dependent reduction in ovarian cysts, with the 50 mg/kg body weight group scoring 2 and the 100 mg/kg body weight group scoring 1. The scoring system was defined as follows: score 0 indicated the absence of lesions, score 1 indicated less than 30% occurrence, score 2 indicated between 30% and 50% occurrence, and score 3 indicated more than 50% occurrence of lesions.

To identify ovarian alterations, our findings revealed that the PCOS group’s ovaries exhibited numerous larger cysts and enhanced vacuolar degeneration. In line with the findings of previous studies [29, 30], the decreased number of follicles reflected androgen overproduction, which controlled the normal follicular maturation process in the PCOS group [30]. In contrast, the marjoram groups demonstrated remarkable recovery of the ovarian tissue and a noticeable reduction in cysts. The harmful effects of DHEA on ovarian tissue were corrected by marjoram extract, probably because of its antioxidant and anti-inflammatory properties.

### Characterization of active metabolites derived from marjoram extract using UHPLC-MS

The base peak chromatograms for marjoram extract metabolites were represented in Figure S1. The annotation of these metabolites was determined based on their retention times in comparison to external standards, as well as tandem mass spectra (including quasi-molecular ions and characteristic MS/MS fragmentation patterns) and illustrated in Table S1. This was further supported by consulting reference literature and the Dictionary of Natural Products (DNP) database, which offers a comprehensive open-access resource on the structural information of a wide array of natural compounds, thereby ensuring a high confidence level in the annotation process.

As mentioned in Table S1, 70 metabolites were annotated in both negative and positive modes, including flavonoids, phenolic acids, mono and diterpenes, quinones, aliphatic acids and fatty acids.

### Annotation of compounds absorbed in rat plasma following marjoram administration

To identify and structurally characterize the bioactive components of marjoram extract absorbed in vivo, a serum metabolomics approach using UPLC-MS/MS was employed to chemically profile the serum collected post-marjoram administration. The resulting base peak intensity chromatograms (BPCs) of all samples are displayed in Figures S2-S4. Additionally, the MS data collected, encompassing retention times, accurate molecular masses, and fragmentation patterns, alongside information from the marjoram extract, published literature, and authentic references, were employed for the structural identification of the absorbed compounds. Through this analysis, a total of 25 compounds; 8 prototypes and 17 metabolites derived from marjoram extract were identified (Table 2), by comparing serum samples from the PCOS group with those treated with the extract. The compounds identified were categorized into various classes based on their structural characteristics, such as aliphatic acids, flavonoids, origanines, phenolic acids, fatty acids, lignans, and monoterpenes. The prototype compounds, represented by peaks (1, 2, 3, 4, 5, 6, 7 and 8), were definitively characterized by cross-referencing UPLC-MS/MS data with results from the marjoram extract analysis as fumaric acid, thymohydroquinone, gastrodin, origanine B/C, caftaric acid, apigenin, sebacic acid and oleic acid, respectively. Furthermore, a tentative identification of 18 metabolites comprising of 2 aliphatic acids, 3 phenolic acids, 1 monoterpene, 2 origanines, 2 lignans, 5 fatty acids, and 3 flavonoids was made based on marjoram extract data, accurate mass measurements, and the molecular weight differences between detected metabolites and their prototype structures, following the principles of in-vivo drug metabolism.

**Table 2:** Characterization of in vivo absorbed metabolites of marjoram extract in rats serum

No.	row m/z	row retention time	Ion type	Elemental composition	Metabolite name	Fragmentation	Transformation	References
M1	311.06	0.77	[M + H]^+^	C_10_H_14_O_11_	Malic acid glucuronide (isovaleryl glucuronide)	195.04, 117.01, 135.02	Glucuronidation	[28, 32]
M2	226.07	0.78	[M + H]^+^	C_10_H_11_NO_5_	Vanillic acid L-glycine (Vanilloylglycine)	170.05, 151.03	Glycine conjugation	[28]
M3	322.14	0.87	[M-H]^−^	C_16_H_21_NO_6_	Caffeic cid L-carnitine	197.03, 135.04	Carnitine conjugation	[28]
M4	261.23	1.02	[M-H]^−^	C_9_H_8_O_7_S	Caffeic acid 4-O-sulphate	242.99	Sulfonation	[28]
M5	172.91	1.03	[M-H]^−^	C_6_H_7_NO_5_	Fumaric acid L-glycine	115.03, 71.01	Glycine conjugation	[28]
1	117.03	2.13	[M + H]^+^	C_4_H_4_O_4_	Fumaric acid	115.03, 71.01	Prototype	[28, 32]
2	165.04	5.54	[M-H]^−^	C_10_H_14_O_2_	Thymohydroquinone	151.31	Prototype	[32, 33]
M6	690.58	5.78	[M-H]^−^	C_31_H_33_NO_17_	Origanine L-glycine (Vandateroside II)	589.15, 633.14	Glycine conjugation	[35]
M7	327.35	5.89	[M + H]^+^	C_16_H_22_O_7_	Thymol-*O*-glucouronide	149.09, 151.11, 97.05, 93.06, 81.06, 43.05	Glucuronidation	[34]
M8	441.17	6.52	[M-H]^−^	C_20_H_24_O_9_S	lariciresinol sulphate	359.1, 343.15	Sulfonation	[29]
M9	376.14	7.48	[M-H]^−^	C_20_H_24_O_7_	7-hydroxyLariciresinol (Tanegool)	359.16, 343.15	Hydroxylation	[29]
M10	378.15	9.04	[M-H]^−^	C_16_H_26_O_10_	Sebacic acid glucuronide	201.04, 183.10, 113.09	Glucuronidation	[28]
3	287.29	9.33	[M + H]^+^	C_13_H_18_O_7_	Gastrodin	145.04, 107.04, 163.06, 125.05	Prototype	[11]
4	815.70	12.77	[M + H]^+^	C_38_H_38_O_20_	Origanine B/C	571.14	Prototype	[35]
5	313.24	13.18	[M + H]^+^	C_13_H_12_O_9_	Caftaric acid	149.03	Prototype	[28]
M12	625.51	15.63	[M + H]^+^	C_27_H_28_O_17_	Quercitrin-*O*-glucuronide	449.10, 303.05, 431.09, 285.03	Glucuronidation	[11, 28]
M13	421.32	16.06	[M-H]^−^	C_25_H_43_NO_4_	Linolenic acid L-carnitine	279.01, 261.31, 54.00	Carnitine conjugation	[31]
M14	338.27	16.26	[M + H]^+^	C_20_H_35_NO_3_	Glycine linoleamide	281.00, 280.31, 202.01, 54.00	Glycine conjugation	[31]
M15	340.52	18.40	[M + H]^+^	C_20_H_37_NO_3_	Oleoylglycine	283.01, 265.31, 247.03, 54.00	Glycine conjugation	[31]
M16	289.00	20.50	[M-H]^−^	C_15_H_14_O_6_	Catechin	289.01	Dealkylation	[11, 28]
M17	319.29	22.58	[M + H]^+^	C_16_H_14_O_7_	methoxy taxifolin (Dihydroisorhamnetin)	301.07	Catechol O-methylation	[11, 32]
6	271.25	22.77	[M + H]^+^	C_15_H_10_O_5_	Apigenin	151.00, 225.05, 119.04	Prototype	[28, 36]
7	201.24	23.11	[M-H]^−^	C_10_H_18_O_4_	Sebacic acid	183.10, 113.09	Prototype	[28, 32]
M18	297.45	23.18	[M-H]^−^	C_18_H_34_O_3_	16-hydroxyoctadec-9-enoic acid	168.09, 54.00	Hydroxylation	[32]
8	283.47	26.40	[M + H]^+^	C_18_H_34_O_2_	Oleic acid	263.09, 237.11, 54.00	Prototype	[31, 32]

Primarily, the phase I metabolic pathways include reduction, hydration, methylation, dehydration, hydroxylation, de-alkylation and acetylation. However, the principal metabolic pathways for phase II involve glucuronidation, S-cysteine conjugation, and glycine conjugation [31]. The absorbed compounds in the serum were numbered according to their order of elution.

#### Phenolic and aliphatic acids characterization

The metabolite M1 exhibited MS/MS fragment at m/z 135.02, indicative of malic acid, and at m/z 311.22 pointing to a glucuronidation process with the addition of a glucuronic acid unit (around 176 Da). The presence of fragmentation ions at m/z 117.01 indicate loss of water molecule (-18 Da).

The initial ion at m/z 179.15 (M-H) was characteristic of a caffeic acid component (Table 2), while the ion at m/z 261.23 hinted at the incorporation of a sulphate unit to give M4 metabolite (caffeic acid-4-O sulphate). The attachment of a carnitine moiety, resulting in an increase of 143.2 Da, was evidenced by fragments such as m/z 322.22 for M3 (caffeic cid L-carnitine), with other fragments suggesting possible losses related to the sulphate and carnitine groups, alongside additional molecular degradation. Furthermore, the precursor ion at m/z 226.12 for M2 metabolite was suggestive protonated vanilloylglycine, and another precursor ion at m/z 170.05 indicated loss of glycine group, resulting in a decrease of approximately 56 Da.

The precursor ion at m/z 115.06 was suggestive of deprotonated fumaric acid, and another precursor ion at m/z 172.91 for M5 metabolite indicated glycine conjugation, adding about 56 Da. This conjugation was reflected in the fragment ions, which depicted the core structure of fumaric acid and elements of the glycine molecule as reported by Taamalli et al., 2015.

#### Lignan characterization

The parent ion of (+)-lariciresinol is observed at m/z 359.10 (Table 2). This ion represents the deprotonated form of lariciresinol ([M-H]^−^). Meanwhile, C_20_H_24_O_9_S has a primary molecular ion indicated by m/z 441.12 for M8 metabolite. This mass reflects the addition of a sulphate group (SO_4_) by adding 82 Da, and fragment at m/z 343.15 suggesting further fragmentation, possibly through the loss of water (H_2_O, 18 Da) from the m/z 361.16 ion, indicating a common pathway in the degradation of phenolic compounds. Also, C_20_H_24_O_7_ shows a primary molecular ion at m/z 376.14 ([M-H]^−^). The fragment ions at m/z 359.16, could be attributed to the loss hydroxyl group (16 Da). The fragment at m/z 343.1545 likely results from further loss of water (OH, 17 Da), highlighting a common fragmentation route for 7-hydroxylariciresinol (M9 metabolite) [32].

#### Flavonoids characterization

Flavonoids represent a significant category of polyphenolic compounds, known for their anti-inflammatory, antioxidant, antitumor, and immunomodulatory effects. Previous studies have highlighted that glucuronidation, sulfonation, and methylation play essential roles in the metabolic pathways of flavonoids [33]. The metabolite Quercitrin-*O*-glucuronide (M12) with molecular formula C_27_H_28_O_17_ showed molecular ion is at m/z 625.14, which is characteristic for protonated Quercitrin-*O*-glucuronide ([M + H^+^]). The fragmentation ions at m/z 449.10 suggests the loss of a glucuronic acid moiety (approximately 176 Da) from the molecular ion. Moreover, the fragment at m/z 431.09 likely results from the loss of water (H_2_O, 18 Da) from the m/z 449.10 fragment, a common occurrence in the fragmentation of polyphenolic compounds [34], while other fragments at m/z 303.05 and 285.03 could represent further breakdown of the flavonoid core, indicating cleavage of additional glucuronic acid linkages and subsequent loss of small molecules like water or carbon dioxide. Also, the catechin base molecular ion showed at m/z 289 for M16 metabolite indicated a loss of methyl group (15 Da) from the parent compound 3-*O*-Methyl-catechin (meciadanol) found in marjoram extract (Table S1) at retention time 25.51 min in deprotonated form ([M-H]^−^) and has an m/z of 303.28. The molecular formula C_16_H_14_O_7_ for M17 metabolite, indicating an additional methyl group compared to the parent taxifolin. The detected ion at m/z 319.29 corresponds to methoxy taxifolin (dihydroisorhamentin) metabolite. Apigenin was identified in its unaltered form along with its fragments. The fragment at m/z 271.25 is likely the protonated molecular ion of apigenin ([M + H]^+^). Furthermore, the fragments at m/z 225.05 indicate a reduction of carbonyl group (-44 Da), while fragmentation ions at m/z 158.90 and 151.00 indicate a deeper decomposition of the apigenin structure, potentially due to the breaking of the aromatic ring or the removal of various functional groups, reflecting significant alterations or the loss of elements from the core flavonoid structure [34].

#### Fatty acids characterization

Fatty acids were classified as unsaturated fatty acids metabolites which were represented in peaks M13, M14, M15, M18 and 8, and saturated fatty acids that were presented in peaks M10 & 7. The mass fragmentation of saturated fatty acids is represented by [M-H-CO_2_] - and [M-H-H_2_O]- fragments in addition to the characteristic fragment at 54 m/z that result double-bond transfer and α-cleavage [35]. Based on MS, MS/MS data in addition to literature as well as marjoram constituents MS/MS mentioned in Table S1 as peaks 59, 62, 66, 67, 68, 70 that were tentatively annotated as oleanolic acid, linolenic, linoleic acid, hydroperoxyoctadecadienoic acid, sebacic acid and oleic acid, respectively. In Table 2 M10 metabolite was tentatively annotated as sebacic acid glucuronide as m/z 378.15 represents the deprotonated molecule of sebacic acid glucuronide and fragment at m/z 201.04 could result from the cleavage of the sebacic acid portion from the glucuronide moiety. M13 metabolite showed a fragment at m/z 421.32 that represents the deprotonated molecule of linolenic acid L-carnitine. This ion indicates the whole molecule minus one hydrogen atom, while fragment at m/z 279 likely represents the linolenic acid moiety after it has been cleaved from the L-carnitine portion. Linolenic acid has a molecular weight approximately in the range of 278 Da (C_18_H_30_O_2_), which aligns with this fragment, suggesting the loss of the L-carnitine part and possibly minor adjustments like the loss or movement of hydrogen atoms during the fragmentation process, also another metabolite for linoleic acid was identified as Glycine linoleamide (M14 metabolite) with m/z 38.27 represents the protonated form and m/z 281 and 280 suggesting the loss of the glycine part. Oleic acid was found unchanged with its metabolite as oleoylglycine in peaks 8, M15, respectively.

Finally, a quasi-molecular ion observed at m/z 690.58 and m/z 589.15 in negative ion mode led to the suggestion of C_31_H_33_NO_17_ for M6 metabolite as its molecular formula suggest as a glycine conjugated form of parent origanine A, by increasing 56 Da for glycine conjugation compared with marjoram extract constituents and previously reported literature [36].

### Network pharmacology analysis

The 25 absorbed compounds in the analyzed serum samples were identified as potential bioactive constituents of the extracts under investigation. Subsequently, these compounds were employed to foresee possible targets and pathways associated with marjoram extract in the context of PCOS, using network pharmacology approaches. The connections between these compounds and their potential therapeutic targets were visualized and scrutinized through a component-target network constructed using Cytoscape 3.7.1.

#### Identification of PCOS-associated target genes of marjoram constituents via network pharmacology-based analysis

To identify the target genes related to PCOS in marjoram compounds, a compound-target network was developed based on search results from the STITCH 5.0 database and SEA search server. STITCH is an extensive database detailing the chemical interactions, encompassing data on over 68,000 chemicals, of which 2,200 are drugs. It links these chemicals to 1.5 million genes across 373 genomes [37].

**Fig. 2:**
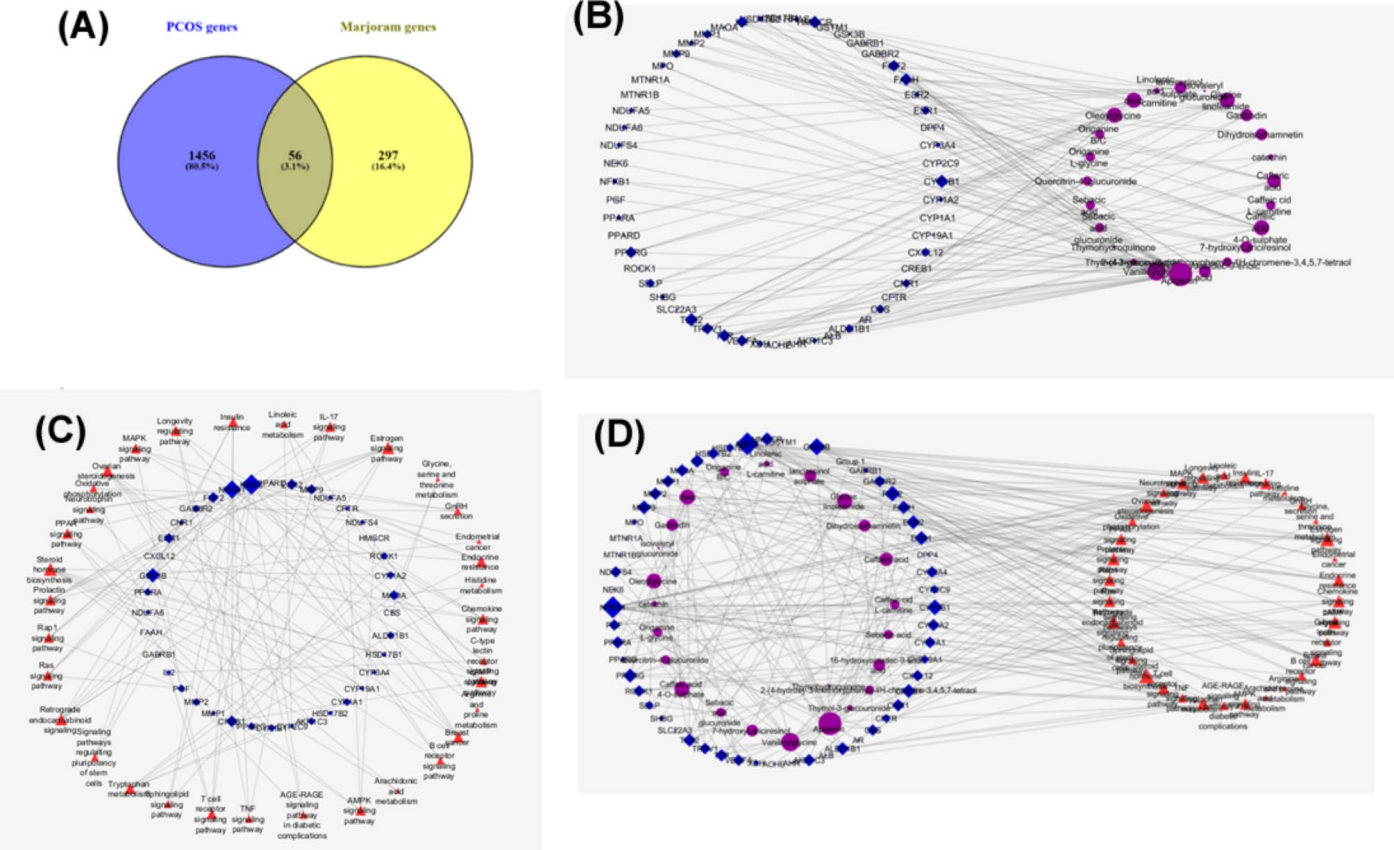
(**A**) Venn diagram of marjoram and PCOS genes and (**B**) components-targets network, The nodes represent targets (blue hexagons), compounds (purple circles) and pathways (red triangles). The edges were regarded as the association between the nodes. Enrichment of nodes are indicated by node size. (**C**) gene-pathway network and (**D**) component-targets-pathway network. The nodes represent targets (blue hexagons), compounds (purple circles) and pathways (red triangles). The edges were regarded as the association between the nodes. Enrichment of nodes are indicated by node size

The 25 absorbed compounds of marjoram, along with their predicted targets sourced from the STITCH 5.0 database, resulted in a total of 735 targets. From GeneCards, 1512 PCOS-related target genes were identified, and by aligning the genes from the compounds with those associated with PCOS, 56 overlapping druggable target genes related to the active components of marjoram were discovered (as shown Fig. 2.A and Table S2). Remarkably, 24 out of the 25 marjoram metabolites demonstrated interactions with PCOS-related targets, including apigenin being oleic acid, vanilloyl glycine and glycine linoleamide and caffeic acid-4-O-sulphate. Natural compounds, including phenolic compounds and flavonoids, have demonstrated significant efficacy in combating PCOS. Notably, apigenin, a widely consumed phytoestrogen flavonoid found in fruits and vegetables, plays a crucial role in enhancing the hormone levels of the pituitary-ovarian axis in PCOS, owing to its potent antioxidant and anti-inflammatory properties [38]. Furthermore, apigenin has been shown to normalize hormonal imbalances, lipid profiles, and antioxidant levels in PCOS [39]. Caffeic acid has been effective in correcting irregular estrous cycles, reducing fasting blood glucose, and improving lipid profiles in PCOS rats induced by DHEA. It also reduces hyperandrogenism, boosts the expression of steroidogenesis enzymes, and alters the expression of proteins related to apoptosis [40]. Additionally, oleic and linoleic acids, which are polyunsaturated fatty acids (PUFAs), have proven beneficial in regulating menstrual cycles, promoting ovulation, reducing hirsutism, lowering inflammation, and enhancing insulin sensitivity. These actions collectively contribute to mitigating other PCOS-related issues such as obesity, diabetes, cardiometabolic diseases, and infertility [41].

#### PPI network

The STRING analysis revealed that the genes with overlap were closely linked in the protein-protein interaction network (Figure S5). This network comprised 56 nodes, each representing a biologically significant target protein, and 325 edges that depicted interactions between proteins. The average degree of connectivity per node was 11.6. It has been observed that marjoram demonstrates its therapeutic effects on PCOS by influencing 56 intersecting target genes. Remarkably, the core components of the protein-protein interaction network were cytochrome P450 family 1 subfamily B member 1 (CYP1B1), toll-like receptor 2 (TLR2), fatty acid amide hydrolase (FAAH), peroxisome proliferator-activated receptor gamma (PPARG), transthyretin (TTR), and 3-Hydroxy-3-Methylglutaryl-CoA Reductase (HMGCR), highlighting their significant roles as therapeutic targets for PCOS, upon which marjoram could potentially exert its effects.

Aromatase, an enzyme critical to steroidogenesis and a member of the complex Cytochrome P450 family, is pivotal in converting steroids, especially in transforming androgens to estrogens. A lack of aromatase disrupts this process, leading to a stop in conversion and adversely affecting ovarian function by raising androgen levels due to the unconverted C19 to C18. Within PCOS research, the aromatase gene, CYP1B1, has been highlighted as crucial [42]. Additionally, increased expressions of TLR2 protein and other pro-inflammatory mediators have been observed in PCOS, contributing to oxidative stress in the condition [43].

The endocannabinoid system, integral to various biological functions, comprises cannabinoid receptors, endocannabinoids like Anandamide (AEA) and 2-arachidonoylglycerol (2-AG), and enzymes for their metabolism. AEA is degraded by fatty acid amide hydrolase (FAAH), and 2-AG by monoacylglycerol lipase. This system plays a vital role in the reproductive organs of mammals [44].

#### Compound-target- pathway network

**Fig. 3:**
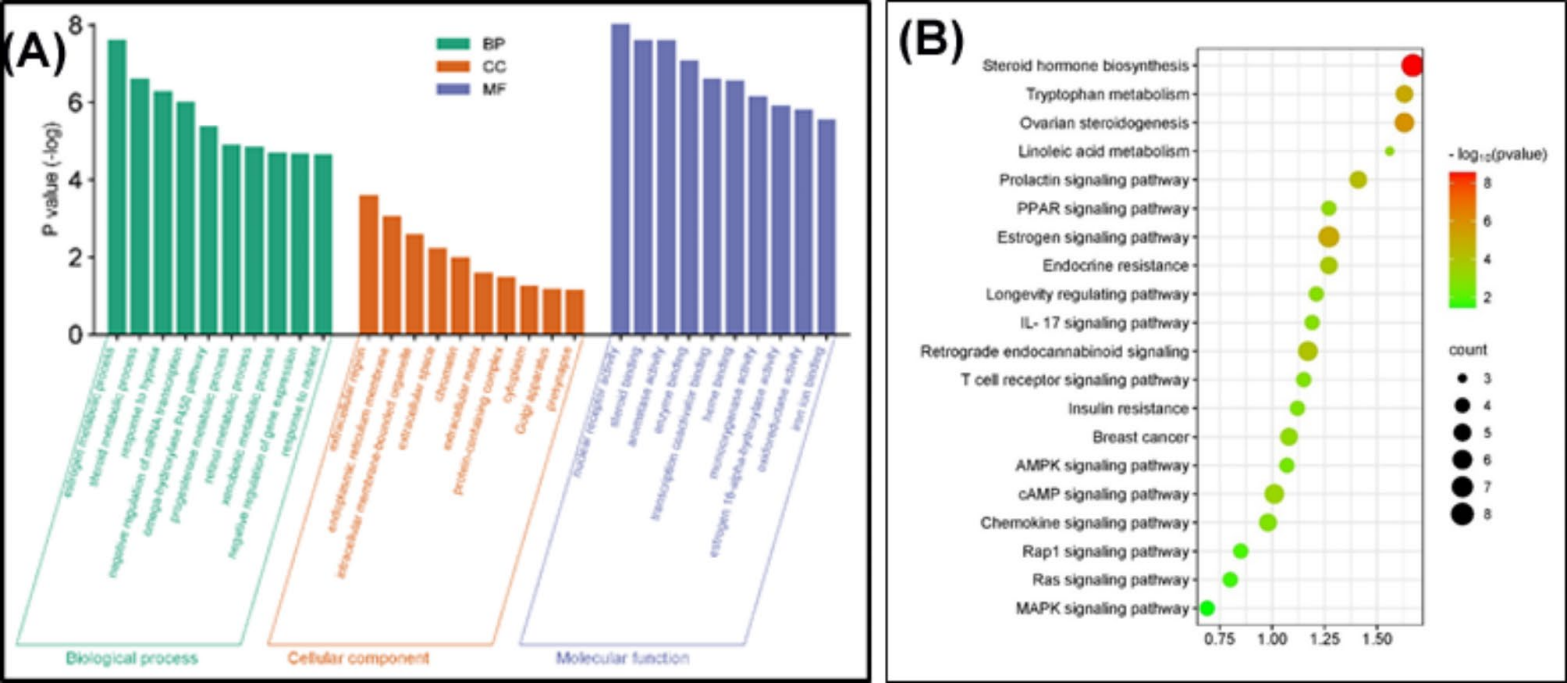
(**A**) GO terms related with marjoram’s overlapping potential targets in PCOS therapy. The top ten functional categories for molecular function, cellular components, and biological processes were selected. (**B**) Bubble chart of top 20 KEGG signaling pathways related to the effect of marjoram against PCOS

The use of Cytoscape software enabled the development of networks for component-target (Fig. 2.B.) and gene-pathway (Fig. 2C.) interactions, which were subsequently merged into a “component-gene-pathway network”, as depicted in Fig. 3.B. This illustrates the complex, multi-component, multi-target, and multi-pathway strategies of marjoram for treating PCOS. Figure 2.B-D demonstrate the interactions between components and target genes, featuring a network with 80 nodes which divided into 24 components and 56 genes and 124 edges, indicating an average of 3.1 targets per component, highlighting the compounds’ multi-target properties. Additionally, KEGG pathway analysis showed that the 38 identified targets participate in 34 pathways related to PCOS, as shown in Fig. 2D.

Using the network analyzer built into CytoScape 3.7.1, the topological parameters of the PCOS network treated with marjoram were examined in order to pinpoint the core targets and constituents. The most active components of PCOS impacted by marjoram, as indicated in Table S3, are apigenin, vanilloylglycine, glycine linoleamide, oleic, oleoylglycine, and caffeic acid 4-O-sulphate. The chemical components in the network with a relatively high degree of connectivity have a tendency to interact with most of the targets in the network, which could be the main pharmacodynamic basis for marjoram’s therapeutic effects when treating PCOS. Likewise, the proteins with the highest median values in the network are CYP1B1, TLR2, TTR, HMGCR, and FAAH, suggesting that they interact with numerous constituents (Table S4). These findings highlight their significant roles as therapeutic targets for PCOS, upon which marjoram could potentially exert its effects.

After merging the previously described interaction networks, a comprehensive “multi-components-multi-targets-multiple pathways” network was constructed, as shown in Fig. 2D. This network enabled the simultaneous identification of synergistic compounds derived from the studied marjoram, systematically uncovering the key molecular targets and exploring how the main components regulate various signaling pathways. This integrated approach provides a detailed understanding of the biological mechanisms through which marjoram bioactives contribute to the management of PCOS, clearly demonstrating that marjoram operates through a holistic approach that involves multiple targets and pathways to exert its pharmacological effects.

#### Gene ontology and KEGG pathway analysis of marjoram and PCOS

Gene Ontology (GO) categorizes gene products into three independent categories: biological process, cellular component, and molecular function. Each query sequence can be assigned multiple GO terms [37]. GO enrichment analysis of 56 target genes was performed using DAVID bioinformatics resources, with a focus on Homo sapiens annotations. DAVID is a web-accessible tool that integrates functional genomic annotations with intuitive graphical summaries, rapidly annotating and summarizing lists of gene or protein identifiers based on shared categorical data such as gene ontology, protein domains, and biochemical pathways. It aids in interpreting genome-scale datasets, helping transition from data collection to biological insights [45].

From the analysis depicted in Fig. 3A., the most enriched biological processes identified were the estrogen metabolic process, steroid metabolic process, and response to hypoxia. These processes are crucial in the context of PCOS as they involve the synthesis, breakdown, and regulation of steroid hormones like estrogen and androgens. In PCOS, an overproduction of androgens often occurs, disrupting normal ovarian function and leading to symptoms such as hirsutism, acne, and irregular menstrual cycles [46]. Marjoram has been studied for its potential to normalize hormone levels, potentially helping to manage these disruptions in steroid metabolism. The analysis also identified significant molecular functions, including nuclear receptor activity, steroid binding activity, aromatase activity, and enzyme binding activity. The most involved cellular components were the extracellular region, endoplasmic reticulum membrane, and intracellular membrane-bounded organelle.

From the 56 intersecting target genes identified, 96 significant signaling pathways were found in KEGG analysis with *p*-values less than 0.01. Out of these, 34 PCOS-related pathways were selected for further study, including steroid hormone biosynthesis, the estrogen signaling pathway, ovarian steroidogenesis, retrograde endocannabinoid signaling, and the cAMP signaling pathway (Fig. 3B). As shown in Table S5, the steroid hormone biosynthesis pathway stood out as particularly critical in PCOS, evidenced by its lowest *p*-value (2.70E-09) and the highest observed gene count (8).

#### Molecular docking validation

**Fig. 4:**
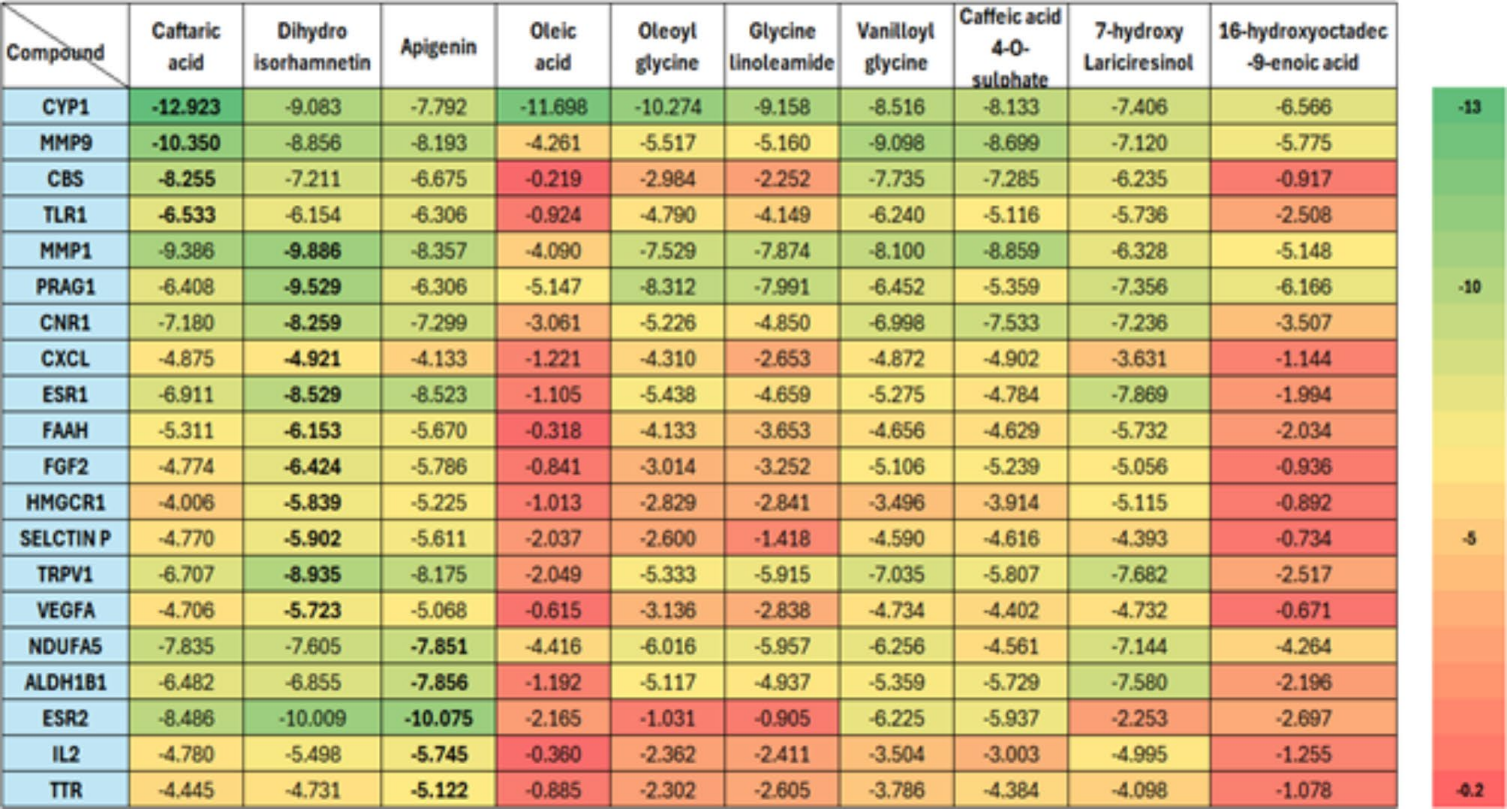
Optimal binding energy of top 20 genes and top 10 marjoram components

The combination of network pharmacology and a systematic pharmacological effect-chemical profiling strategy revealed that the top 20 genes, as listed in Table S4, are highly associated with PCOS. This association is due to their significant connectivity and multiple functional interactions with the top ten active compounds. Consequently, molecular docking simulations were carried out between these essential genes and the leading compounds to confirm their interaction patterns and the effectiveness of the top-ranked constituents with critical target proteins. The simulations showed favorable docking affinities for most of the top ten target compounds with the selected top 20 core target proteins, and the activity levels of these bioactive compounds were assessed and ranked according to their XP Gscore (Fig. 4).

**Fig. 5:**
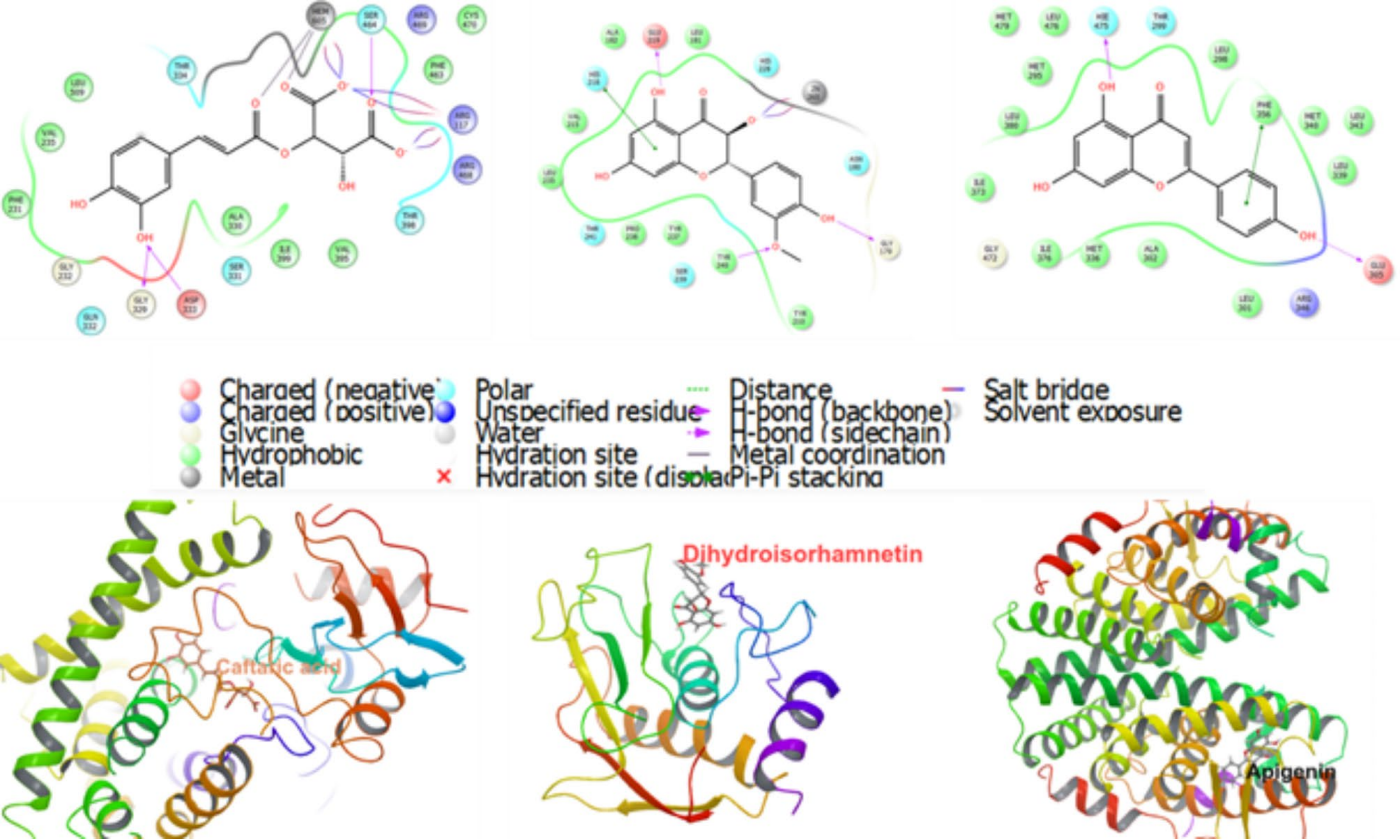
The 2D force and 3D spatial environment between (**A**) Caftaric acid and CYP1B1, (**B**) Dihydroisorhamnetin and MMP1 and (**C**) Apigenin and ESR2

Molecular docking and subsequent binding energy analysis revealed that caftaric acid demonstrated the highest binding affinity towards CYP1B1, MMP9, CBS, and TLR1 with respective binding energies of -12.923, -10.350, -8.255, and − 6.533 kcal/mol, respectively. Additionally, dihydroisorhamnetin exhibited the highest binding affinity for MMP1, PRAG1, CNR1, CXCL, ESR1, FAAH, FGF2, HMGCR1, SELECTIN P, TRPV1, and VEGFA, with binding energies of -9.886, -9.529, -8.259, -4.921, -8.529, -6.153, -6.424, -5.839, -5.902, -8.935, and − 5.723 kcal/mol, respectively. Moreover, apigenin was found to have the highest binding affinity to NDUFA5, ALDH1B1, ESR2, IL2, and TTR, with binding energies of -7.851, -7.856, -10.075, -5.745, and − 5.122 kcal/mol, respectively (Fig. 4). The binding site of caftaric acid within CYP1B1 is depicted in Fig. (5 A). The analysis showed that caftaric acid effectively fits into the active site, achieving the highest Gscore (-12.923 kcal/mol). This binding involves a metal interaction with Hem605, hydrogen bonds with Gly329, Asp333, and Ser464, and positively charged interactions with Arg117, Arg468, and Arg469. Additionally, hydrophobic interactions with Leu509, Val235, Phe231, Ile399, Val395, Phe463, and Cys470, as well as polar interactions with Ser464, Thr398, Ser331, and Thr334 were observed. Dihydroisorhamnetin also demonstrated significant binding affinity, achieving the highest Gscore of -9.886 kcal/mol with MMP1, as shown in Fig. (5B). This interaction involved a metal interaction with Zn265, polar interactions with His228, Asn180, His218, Thr241, and Ser239, hydrophobic interactions with Leu181, Tyr210, Tyr240, Tyr237, Pro238, Leu235, Val215, and Ala182, an H-bond interaction with Gly179, Glu219, and Tyr240, and a negatively charged interaction with Glu219. Moreover, apigenin displayed optimal interaction patterns with ESR2, forming a conformationally fitting complex and achieving a Gscore of -10.075 kcal/mol. As illustrated in Fig. 5C., apigenin formed two H-bonds with Glu305 and His299, hydrophobic interactions with Leu301, Ala302, Met295, and Leu380, a negatively charged interaction with Glu305, and a positively charged interaction with Arg346. These interactions contribute to the formation of a stable complex within the active site of the ESR2 protein, resulting in a high Gscore value (Fig. 4).

These interactions align closely with those documented in the literature [47]. Notably, apigenin exhibited favorable interactions with ESR2, predominantly through hydrophobic contacts that formed a tightly bound complex to ESR2, with a minimized binding energy of -9.802 kcal/mol. It also engaged in hydrogen bonding and polar interactions with active site residues such as Leu346, Thr347, Glu353, Arg394, Leu387, Asp351, Glu419, Cys530, Val534, Leu536, Gly420, and Lys529. These findings substantiate the predicted results, affirming the viability of this integrated approach and providing a reference for systematically uncovering the therapeutic mechanisms of marjoram as a treatment for PCOS.

## Materials and methods

### Chemical and materials

Dehydroepiandrosterone (DHEA), acetonitrile (HPLC grade) were obtained from Sigma-Aldrich Co (St. Louis, MO, USA). Progesterone, estradiol, FSH and testosterone ELISA kits were purchased from (R&D Systems, USA), formic acid was purchased from Fisher Scientific, UK., ultra-pure water produced by a Milli-Q system was used for UPLC analysis, and ethanol was obtained from El Gomherya Pharmaceutical Co., Egypt.

### Herbal sample preparation

2.5 kg of aerial parts of Marjoram (*Origanum majorana* L.), were obtained from the Experimental Station of Medicinal Plants, Pharmacognosy department, Faculty of Pharmacy, Cairo University, Giza, Egypt in January 2023. The plant was authenticated by Prof. Dr. Sania Ahmed, Botany Department, Faculty of Science, Alexandria University, Egypt. A voucher specimen labeled as OM001, has been stored at the Department of Pharmacognosy, Faculty of Pharmacy, Alexandria University, Egypt. A figure for the collected plant specimen is shown in supplementary material (Figure S6). The plant was collected according to the relevant national guidelines. The plant material was cleaned and air-dried in dark room (25 ^◦^C, 60% RH for 2 weeks), then they were ground with an electric grinder.

The shade-dried powdered (1 Kg) aerial parts were extracted with 70% ethanol by ultrasonication, the extracts were collected twice, then combined, evaporated, and stored at − 20 ^◦^C until use.

### Animal study and treatment

6 weeks old post-pubertal Western female Albino rats (No.32) weighing 80–100 g obtained from the National Research Center’s animal house have been utilized in the present investigation. The animals were raised under controlled circumstances (22 5 °C, 55% humidity, and a 12 h light/dark cycle). Animals were provided with access to water and a typical diet. This study’s procedures were conducted in compliance with the standards set by the Research Ethics Committee of the National Research Center’s, as per the approval number 044121223. These procedures were aligned with internationally recognized guidelines for laboratory animal use, adhering to the European Community directives established in 1986 (Directive 86/609/EEC).

#### Experimental design

Proceeding acclimatization for one week, animals were divided into four groups, each included 8 rats:

##### Group I

Control group administered saline orally.

##### Group II

Administered DHEA (SC, 60 mg/Kg BW for one month) dissolved in sesame oil and served as PCOS model [23].

##### Group III

Administered marjoram low dose (50 mg/Kg BW) orally dissolved in distilled water for 1 month post PCOS induction with DHEA [48].

##### Group IV

Administered marjoram high dose (100 mg/Kg BW) orally dissolved in distilled water for 1-month post PCOS induction with DHEA [48].

PCOS was induced in female rats subcutaneously (SC, in the dorsolateral and flank of rats.) via DHEA (60 mg/Kg BW for one month) dissolved in sesame oil. The induction of PCOS was confirmed by hormonal serological tests (Estradiol, progesterone, testosterone and FSH) and histopathological examination (ovary revealed multiple ovarian follicles as cysts) and lastly the increase in body weight in PCOS rats that administered DHEA relevant to control group.

#### Blood sampling and tissue preparation

Rats were weighed and sedated with carbon dioxide. After gathering blood from the retro-orbital vein, samples were centrifuged at 5000 rpm for 10 min for serum separation before being preserved at -80 °C and was kept for biochemical investigations. Rats were sacrificed with cervical dislocation under 75% carbon dioxide anesthesia, and ovarian tissue was isolated and a portion was homogenized in phosphate-buffered saline for further analysis and the other portion was impeded in 10% formaldehyde for histopathological investigations.

#### Measured biochemical parameters

##### Oxidative stress biomarkers

**Serum nitric oxide concentration (NO)**

Nitric oxide was assessed using Randox Company supplied spectrophotometry at 540 nm. Nitrate was transformed to nitrite via nitrate reductase enzyme in acid medium. Nitrite further reacts with Griess reagent to generate an azo compound that can be spectrophotometrically determined at 540 nm [49].

**Serum total antioxidant capacity (TAC)**

Using kits from the Randox Company, the total antioxidant capacity was assessed by measuring the reaction between the sample’s antioxidants and a predetermined quantity of exogenously supplied hydrogen peroxide (H_2_O_2_). A portion of the supplied hydrogen peroxide is removed by the sample’s antioxidants. Through an enzymatic reaction involving the conversion of 3,5-dichloro-2-hydroxy benzensulphonate to a colored product, the residual H_2_O_2_ is measured at 505 nm [50].

##### ELISA determination of serum progesterone, estradiol, FSH and testosterone

Using an enzyme-linked immunosorbent assay kit (R&D Systems, MN, USA) in accordance with the manufacturer’s instructions, the activities of progesterone, estradiol, FSH and testosterone were determined were determined. After that, a quantitative sandwich enzyme immunoassay was used to assess the experiment. Next, the suitable antibodies were pre-coated onto the microplate. Subsequently, the progesterone and estradiol-specific enzyme-linked secondary antibody was added, which was followed by the immobilized antibody. At 450 nm, the absorbance was then determined. The Agient BioTek Microplate reader, Neo2, was used to measure the color intensity at 450 nm [51].

#### Body weight measurements

During the experiment, the rats initial and end body weight was measured. The body weight gain was calculated [52].

#### Histopathological examination

Tissue samples from ovaries were collected from the experimental rat groups, fixed in neutral buffered formalin 10%, washed, dehydrated, cleared and embedded in paraffin. The paraffin embedded blocks were sectioned at 5 micron thickness and stained with hematoxylin and eosin (Bancroft and Gamble, 2008) for histopathological examination. Stained sections were examined by a light microscope (Olympus BX50, Japan).

Histopathological alterations of ovaries were recorded and scored as, no changes (0), mild (1), moderate (2) and severe (3) changes, the grading was determined by percentage as follows: <30% changes (mild change), < 30 – 50% (moderate change), and > 50% (severe change) (Korany et al., 2019).

### Statistical analysis

For the statistical analysis, GraphPad Instat 8 (GraphPad Software Inc., San Diego, CA, USA) was utilized. SPSS 16 was used to analyze the data using one-way ANOVA, followed by a post-hoc Tukey’s test. *P*-values of less than 0.05 were considered statistically significant.

### UHPLC TQD-MS analysis conditions

The analysis of serum samples as well as marjoram alcoholic extract was conducted using a UPLC-TQD-MS/MS system from Waters Corporation, located in Milford, MA 01757, USA. Detailed chromatographic conditions are provided in the supplementary material. Data processing was carried out with Mzmine software (version 2.53), which was employed for importing data, deconvoluting peaks, aligning, and annotating both peaks and spectra, as outlined in [53].

### Metabolites annotation

The apparent annotation of marjoram extract metabolites as well as the absorbed serum metabolites relies on precise mass measurement, retention time analysis, isotopic pattern examination, and MS/MS data. The validity of the acquired mass spectra was confirmed through several methods. Firstly, the retention time and MS/MS spectra were matched against standard metabolites (oleic acid, catechin, fumaric acid and apigenin) to confirm their identity. Secondly, the observed fragmentation patterns were matched against our in-house database and cross-referenced with those documented in the literature. Lastly, the structure information of the metabolites was compared to the data obtained from either the Human Metabolome database (http://www.hmdb.ca/) or the pubchem database (https://pubchem.ncbi.nlm.nih.gov/).

### Network pharmacology analysis

#### Collection of marjoram compounds and corresponding targets

Following the annotation of marjoram extract metabolites and the absorbed compounds in the serum of rats after marjoram extract administration, the chemical structures of the absorbed compounds were obtained from PubChem Compound or created using ChemDraw Ultra 12.0. These structures were then converted into SMILES notations. The molecular structure files were uploaded to the STITCH database, configured for the ‘Homo sapiens’ species (version 5), and SEA search server (https://sea.bkslab.org/jobs/search) to predict interactions with protein targets. The likelihood of interaction between the compounds and each target was determined using the combined score. The protein targets of marjoram were subjected to standardization using the Uniprot protein database.

#### Sorting PCOS-related target genes

Targets associated with polycystic ovary syndrome were identified by conducting a search for “polycystic ovary” in the GeneCards database (https://www.genecards.org), leading to the creation of a disease-target database.

#### Screening common drug and disease targets

The Venny 2.1.0 web application (https://bioinfogp.cnb.csic.es/tools/venny/) was utilized to discover common targets between marjoram and PCOS, which enabled the drafting of Venn diagrams.

#### Functional enrichment and pathway analysis

GO enrichment analysis is an essential computational tool for understanding gene functions, identifying biological processes, cellular components, and molecular functions associated with specific ailments, thus providing deeper insights into gene lists (Dawood et al., 2023). KEGG pathway analysis and GO enrichment analysis were conducted using the functional annotation tool in the Database for Annotation, Visualization, and Integrated Discovery (DAVID) (https://david.ncifcrf.gov/), with “Homo sapiens” selected as the species. Prior to enrichment, gene names were converted to their corresponding UniProt accession numbers, and GO terms with *p*-values < 0.05 were considered statistically significant.

Additionally, data on the Kyoto Encyclopedia of Genes and Genomes (KEGG) pathways and protein-protein interactions (PPI) were collected from the String database (https://string-db.org/), focusing on the “Homo sapiens” species. To ensure high-quality PPI information, a minimum interaction score threshold was set at the highest confidence level of 0.900.

#### Network construction and analysis

Three pharmacological networks were developed as follows: Initially, a compound-target network was established to connect bioactive molecules with their potential protein targets. Following this, a target-pathway network was constructed based on these predicted targets and their disease-related signaling pathways. Finally, an integrated component-target-pathway network was formulated. All three networks were built and visually explored using Cytoscape software (version 3.7.0, http://www.cytoscape.org/, Boston, MA, USA), enabling a comprehensive analysis of the pharmacological effects of marjoram chemical components in the treatment of polycystic ovary from a broad perspective.

In pharmacological networks, the various characteristics of candidate compounds, potential targets, and signaling pathways are represented as different types of nodes, while the interactions among these characteristics are depicted as edges connecting the nodes. Network parameters are assessed using the network analyzer plugin in Cytoscape. The importance of each node within the constructed networks is determined by the degree of contribution, which is based on the number of edges linked to the node. A higher degree value suggests a greater contribution of the node to the subsequent network pharmacology analysis.

#### Molecular docking

To elucidate the pharmacological combinatorial actions of bioactive components identified for PCOS treatment, molecular docking studies were conducted on the top enriched targets from network pharmacology analysis using the Schrodinger Maestro 11.8 software package (LLC, New York, NY). The X-ray crystal structures of the top 20 enriched targets were retrieved from the RCSB Protein Data Bank (http://www.rcsb.org/pdb) and prepared using the protein preparation wizard module in the Schrodinger suite. The selection of crystal structures was based on the best resolution available. During optimization, hydrogen bonds and bond orders were assigned using PROPKA (Jensen Research Group, Denmark), and zero-order bonds to metals and disulfide bonds were created at pH 7.0. Water molecules beyond 5 Å from the binding sites were removed, followed by energy minimization with an RMSD value of 0.3 Å using the OPLS 2005 force field.

The 3D structures of the top ten compounds identified from network pharmacology were imported as SDF files into the LigPrep module of the Maestro 10.2 molecular modeling software package (Schrodinger^®^) to generate low-energy structures. These structures were prepared by generating molecules with correct chiralities, ionization states, stereochemistries, and ring conformations, followed by geometry minimization using the OPLS 3 force field. Ionization at pH 7 was performed to produce all possible states.

The binding sites for the docking studies were defined using the receptor grid generation module, setting the boxes around the centroids of co-crystallized ligands with a size adjusted to ≤ 20 Å. Molecular docking simulations were performed using the Glide 11.8 module (Glide, version 11.8, 2018, Schrödinger, USA) in extra precision (XP) mode, with Gscore as the ranking function. A more negative Gscore indicated a better interaction surface between the compound and protein crystal, as well as stronger activity. The intermolecular interaction patterns between ligands and target proteins in 2D and 3D structures were visualized using the Maestro interface.

## Conclusion

The study in hand investigated marjoram potential in treating PCOS by combining network pharmacology and serum metabolite analysis. It identifies the steroid hormone biosynthesis pathway as crucial for marjoram’s efficacy, spotlighting apigenin and oleic acid as key compounds. This integrated approach underscores marjoram’s rich active ingredient profile, advocating its use in PCOS treatment. The research maps marjoram’s compound interactions, target genes, and pathways, developing a comprehensive model to understand its therapeutic mechanisms. It reveals marjoram’s ability to modulate various signaling pathways, suggesting a holistic pharmacological impact.

The integration of network pharmacology with chemical profiling has identified significant genes associated with PCOS, connected through robust interactions with key compounds such as caftaric acid, dihydroisorhamnetin, and apigenin. These compounds demonstrated high binding affinities and effective docking scores. Specifically, caftaric acid showed the highest affinity for CYP1B1 with a notable binding energy, while apigenin and dihydroisorhamnetin also exhibited strong binding energies towards ESR2 and MMP1, respectively. These results, consistent with existing studies, highlight the potential of these bioactive compounds as effective treatments for PCOS. This synergy of traditional herbal medicine and modern science enhances our grasp of marjoram’s role in PCOS management and its potential as a versatile therapeutic agent, paving the way for further research into its benefits for PCOS and beyond.

## Electronic supplementary material

Below is the link to the electronic supplementary material.

Supplementary Material 1

## Data Availability

Data will be made available upon request.

## Abbreviations

ANOVA: Analysis of Variance
DAVID: Database for Annotation, Visualization, and Integrated Discovery
DHEA: Dehydroepiandrosterone
E2: Estradiol
FSH: Follicle-Stimulating Hormone
GnRH: Gonadotropin-releasing hormone
GnRH: Gonadotropin-Releasing Hormone
GPx: Glutathione Peroxidase
KEGG: Kyoto Encyclopedia of Genes and Genomes
LH: Luteinizing Hormone
MDA: Malondialdehyde
NO: Nitric Oxide
PCOS: Polycystic Ovary Syndrome
PPI: Protein-Protein Interaction
RMSD: Root Mean Square Deviation
SOD: Superoxide Dismutase
SPSS: Statistical Package for the Social Sciences
TAC: Total Antioxidant Capacity
UHPLC-MS/MS: Ultra-High-Performance Liquid Chromatography coupled with Tandem Mass Spectrometry
