# Supplementary material for: Exploring the therapeutic potential of marjoram (Origanum majorana L.) in polycystic ovary syndrome: insights from serum metabolomics, network pharmacology and experimental validation
Source: BMC Complement Med Ther. 2025 Feb 21;25:67. doi: 10.1186/s12906-025-04774-5 (PMC11846456; doi:10.1186/s12906-025-04774-5)
Supplement: Supplementary file 1 — Supplementary Material 1 [file 12906_2025_4774_MOESM1_ESM.docx]

**Exploring the Therapeutic Potential of Marjoram (*Origanum majorana* L.) in Polycystic Ovary Syndrome: Insights from Serum Pharmacochemistry, Network Pharmacology and Experimental Validation**

**Experimental**

**UHPLC TQD-MS analysis conditions:**

The UPLC-TQD-MS/MS was performed using Waters Corporation, Milford, MA01757, U.S.A. the detailed chromatographic condition can be found under supplementary material. The chromatographic separation was performed using the Waters Acquity UPLC BEH C18 column, with dimensions of 50 mm × 2.1 mm ID × 1.7 μm particle size. The column was operated at a flow rate of 0.2 mL.min^-1^ and a temperature of 30°C. The analysis was conducted using a gradient elution method, where the concentration of phase B was increased. A binary mobile phase consisting of 0.1% formic acid in ultrapure water (Phase A) and 0.1% formic acid in acetonitrile (Phase B) was utilized. The sample solution was prepared using UPLC analytical grade methanol at a concentration of 100 μg/mL. The filtration process involved the use of a membrane disc filter with a pore size of 0.2 μm. Prior to injection, the sample was subjected to sonication in order to remove any trapped gases. Subsequently, a volume of 10 μL was injected into the UPLC apparatus. The data acquisition in a mass range of 100-1000 m/z was performed using a Triple Quadrupole (TOD) mass analyzer, employing both negative and positive ionization modes. The working conditions of ESI were optimized by adjusting several parameters included the capillary voltage set at 3 kV, the temperature of the ion source maintained at 150 ^◦^C, the cone voltage set to 30 V, the pressure of the nitrogen gas nebulizer set at 35 psi, and the temperature of the drying and sheath gas set at 440 ^◦^C. The optimal flow rates for the sheath and drying gas were determined to be 50 L/h and 900 L/h, respectively. The duration of the analytical run was increased to 30 minutes. The CID energy was increased incrementally from 30 to 70 eV by introducing nitrogen gas as the collision gas in the second quadrupole collisional cell (Q2). In this study, we employed a third quadrupole mass analyzer (Q3) to assess the daughter ions produced during collision-induced dissociation (CID). Raw LC-MS data were imported into MZmine 2.0 (http://mzmine.sourceforge.net/) for data processing and analysis. The parameters used for processing included a mass range of 100–1000 Da, a retention time (RT) tolerance of 0.1 minutes, and a signal-to-noise threshold of 1.5. A data matrix was then generated, comprising retention time, mass-to-charge ratio (m/z), and peak intensity (Rummun *et al.*, 2023).


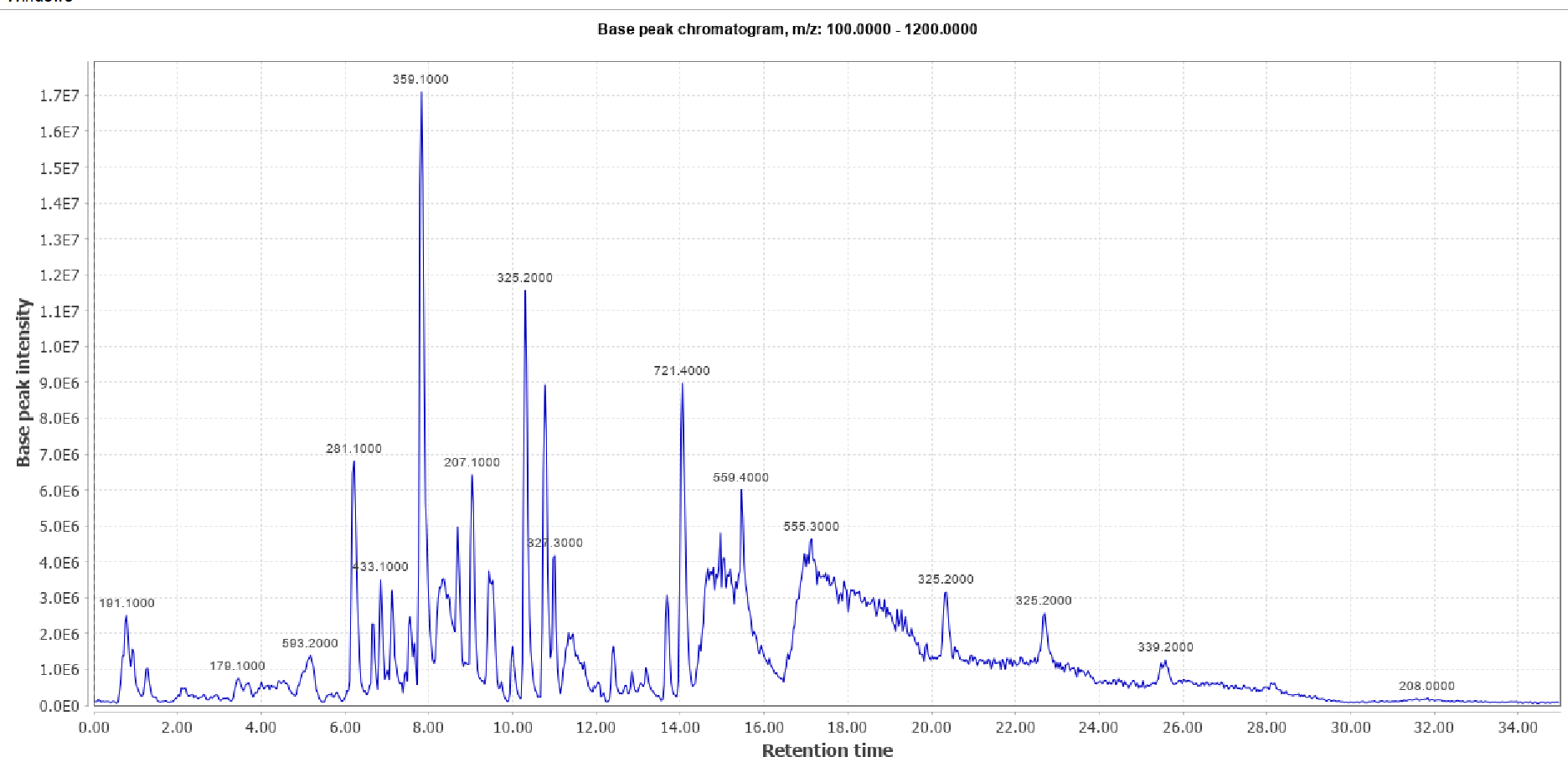


**A**


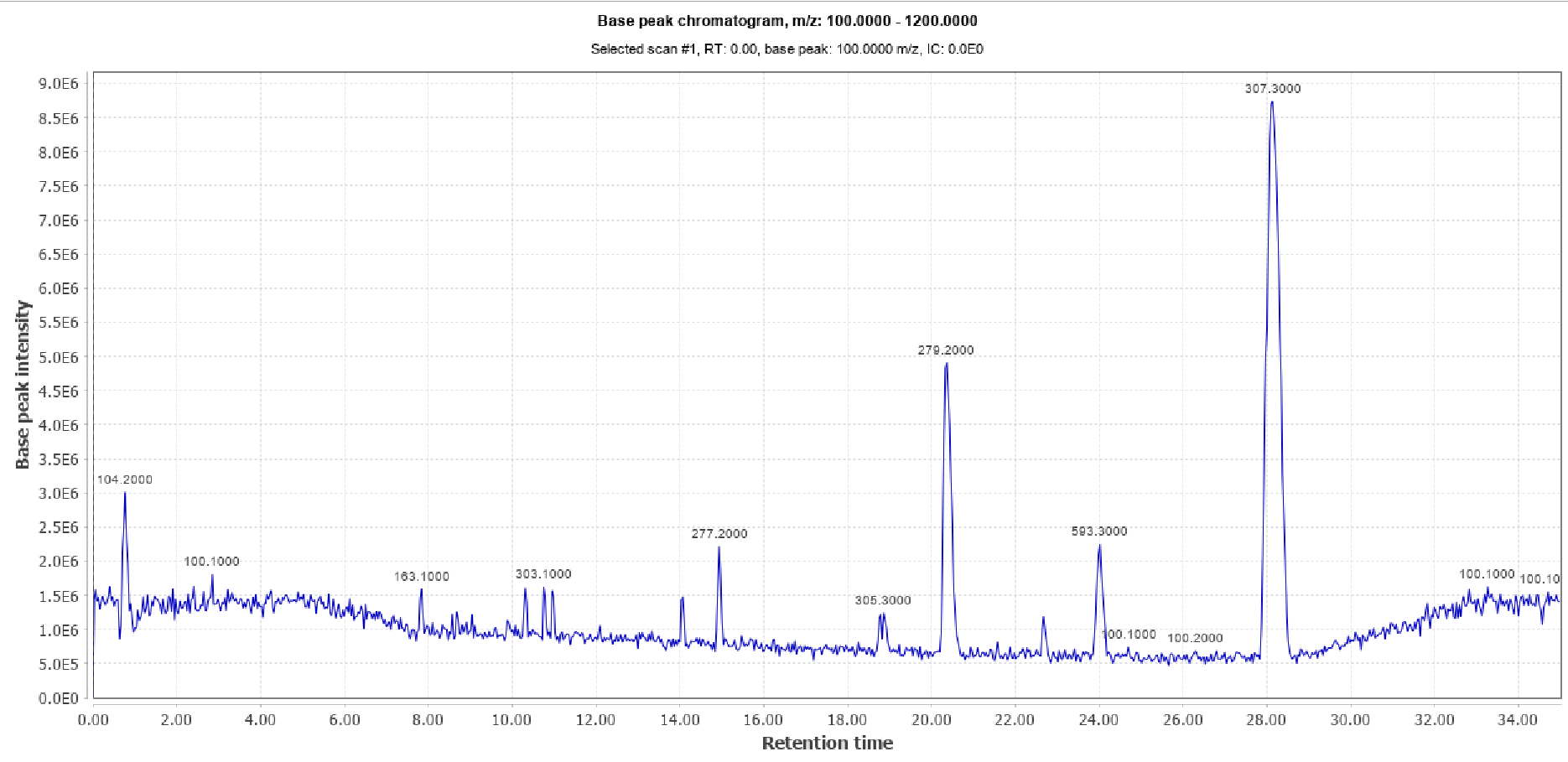


**B**

**Figure S1. Base peak chromatogram of marjoram alcoholic extract on (A) negative ionization mode and (B) positive mode.**


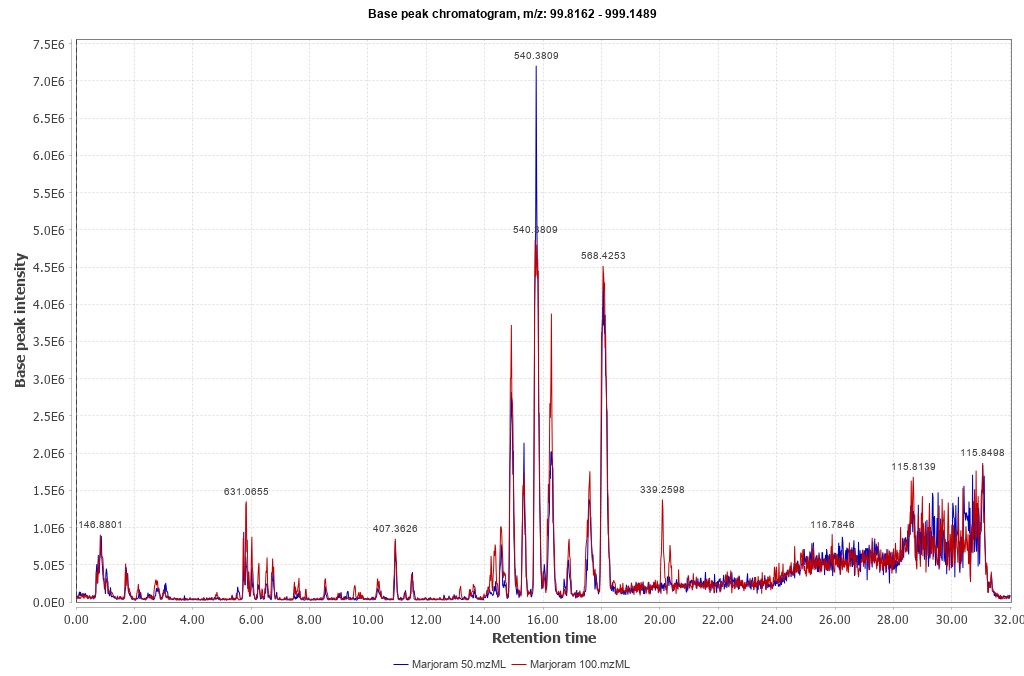


**A**

**Mj100**

**Mj50**


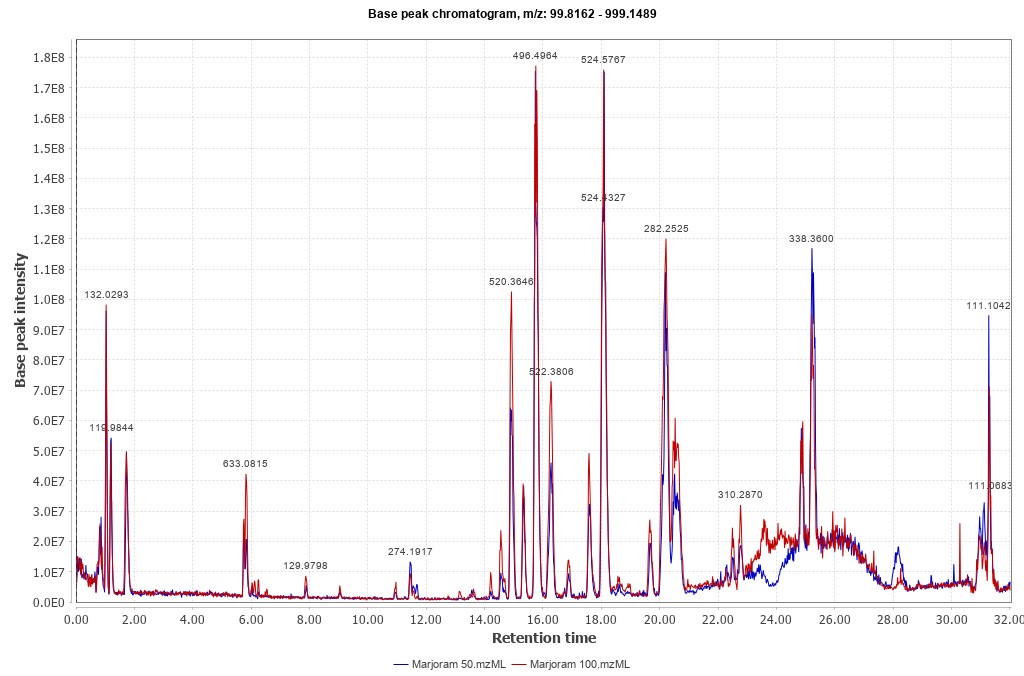
**Figure S2. Base peak chromatogram of dose-based marjoram-treated serum samples in A) negative mode and B) in positive mode.**

**B**

**Mj100**

**Mj50**


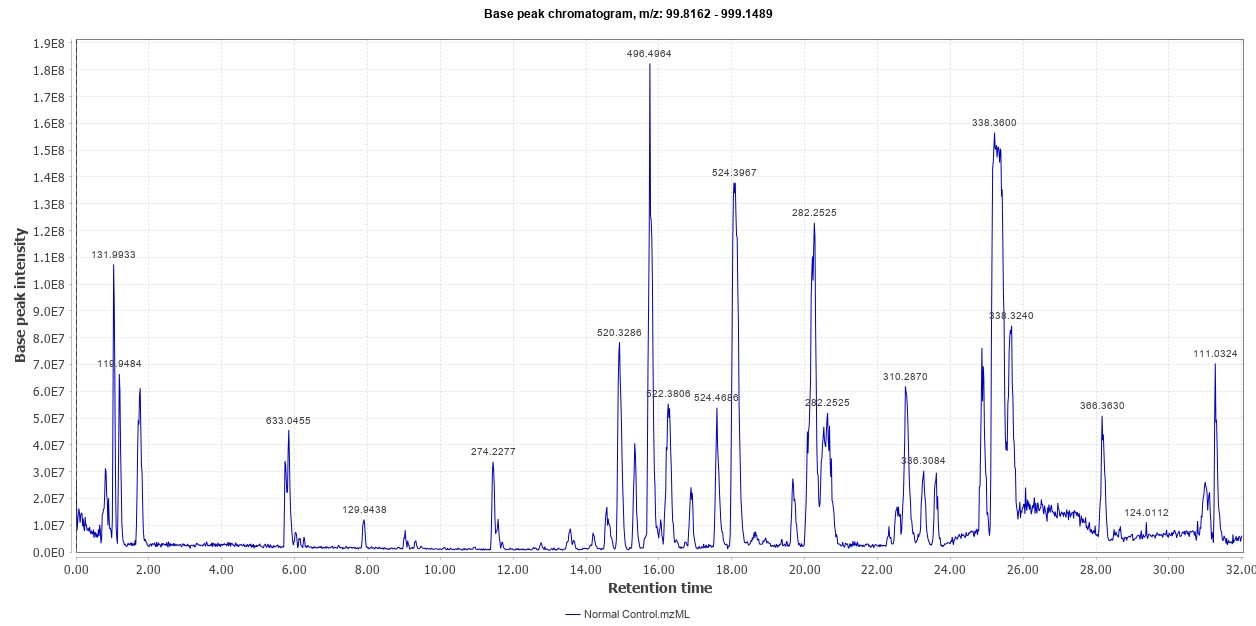

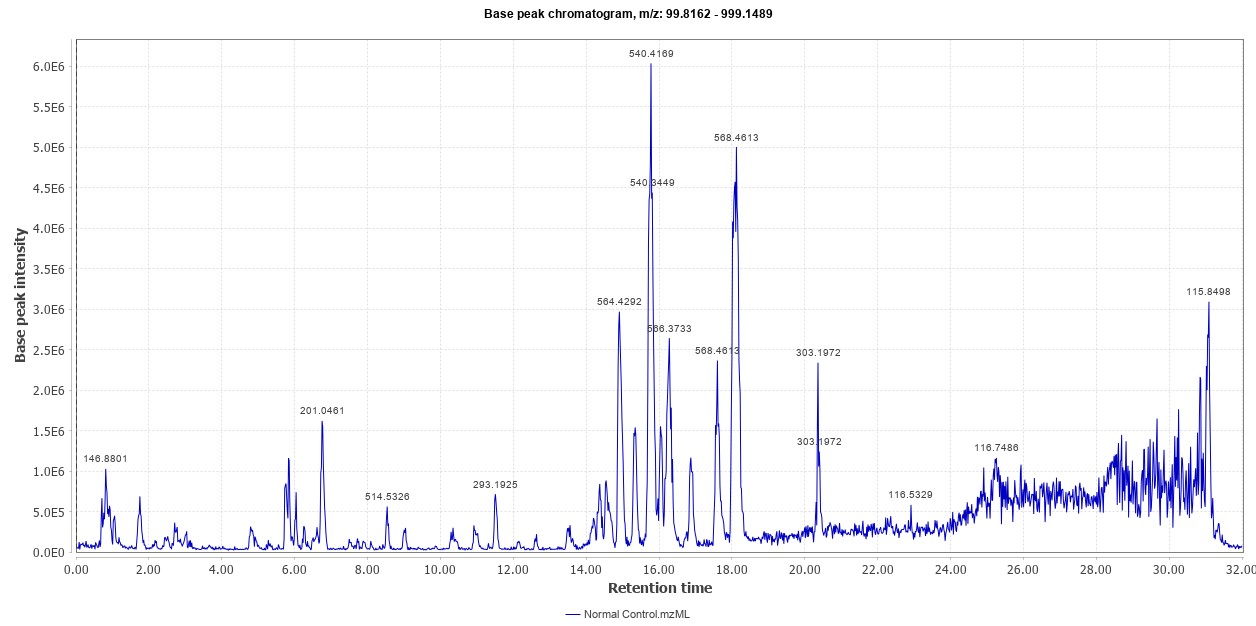


**A**

**B**

**Figure S3. Base peak chromatogram of Control serum samples in A) positive mode and B) negative mode**


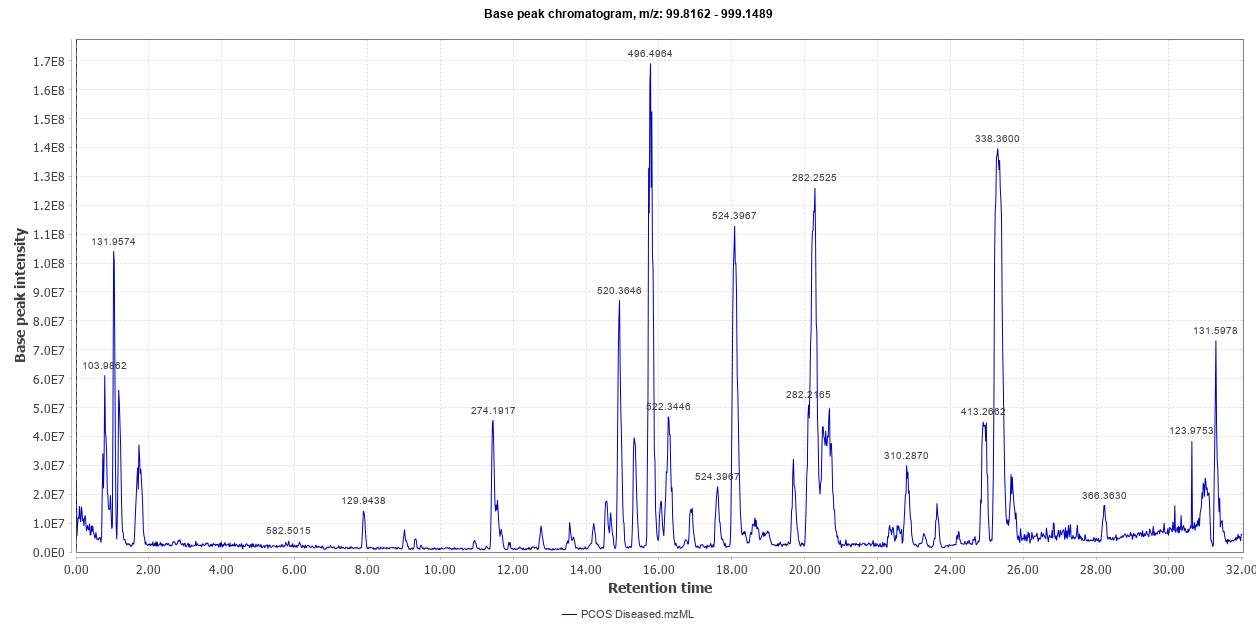


**A**


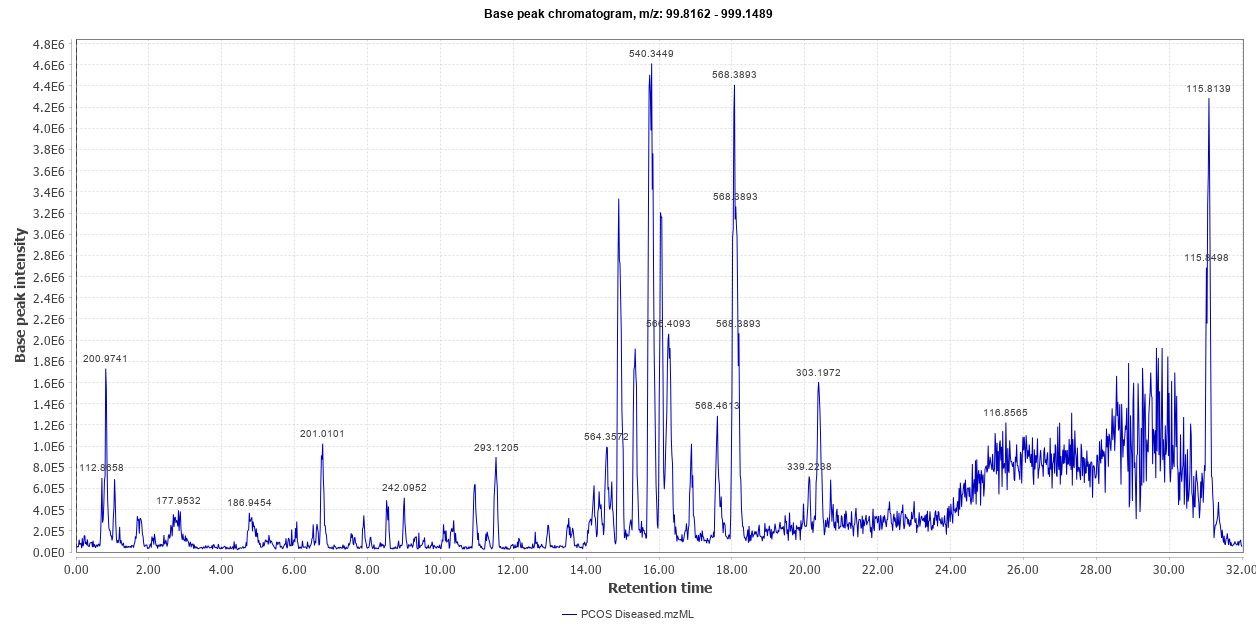
 **Figure S4. Base peak chromatogram of PCOS serum samples in A) positive mode and B) negative mode**

**B**


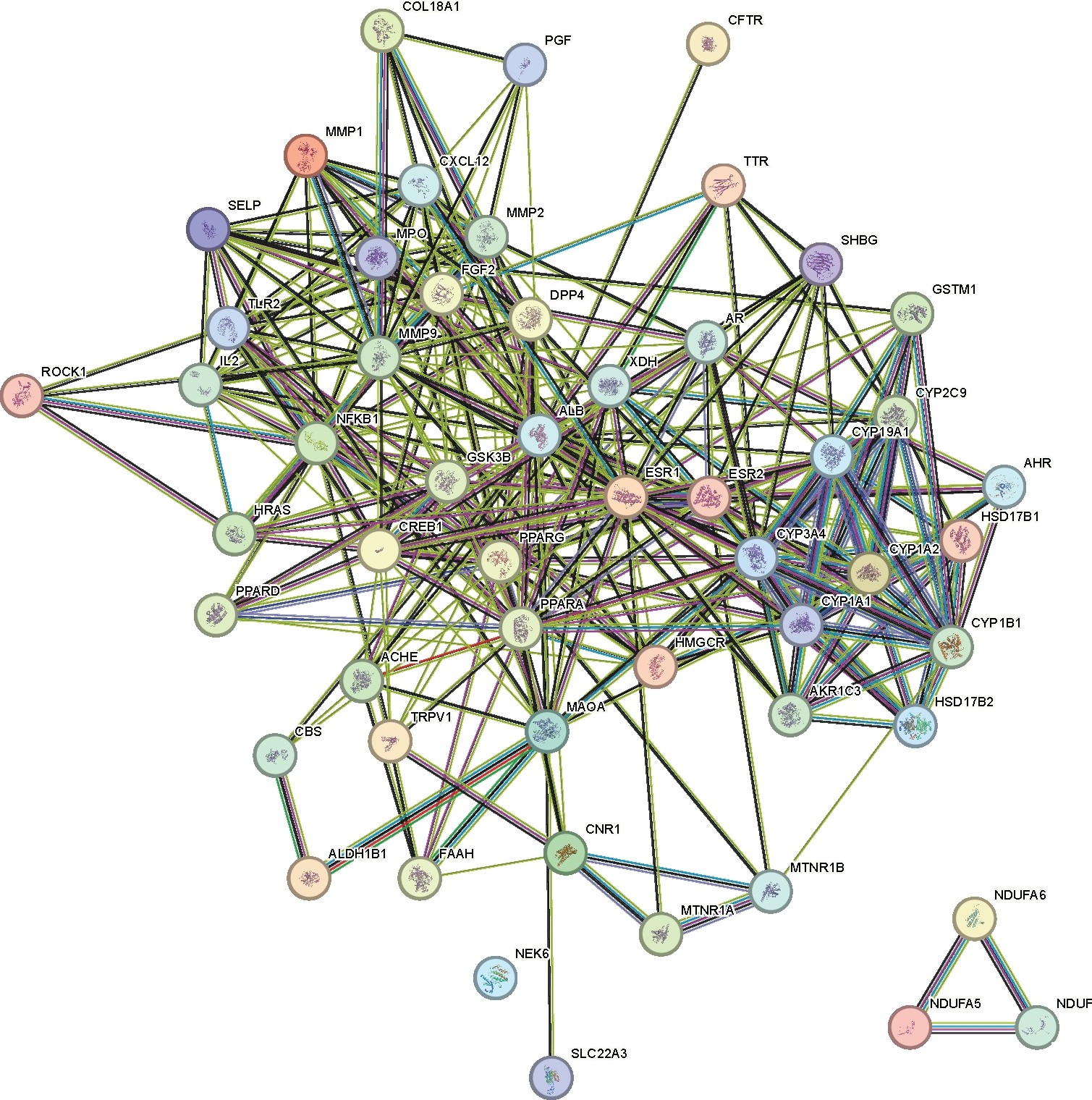


**Figure S5. Protein-protein interaction diagram of associated with marjoram absorbed compounds.**


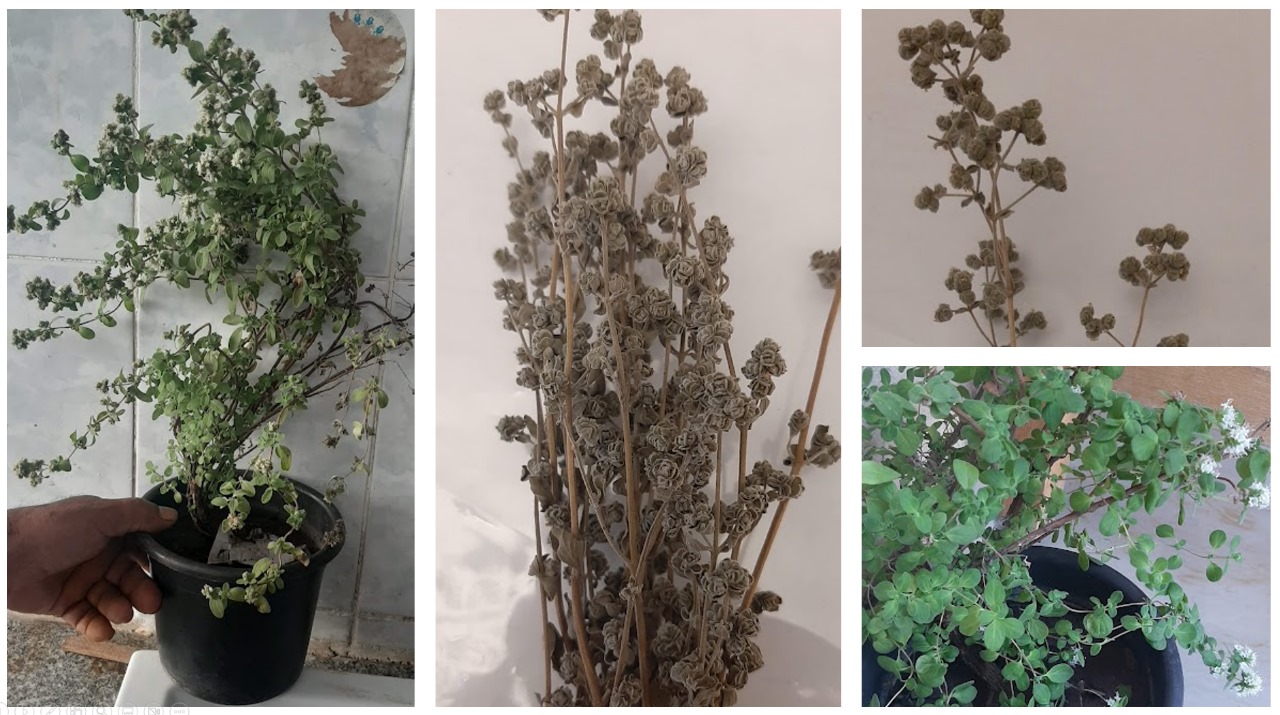


**Figure S6: Origanum majorana L. collected plant specimen**

|  | **Table S1. Characterization of marjoram extract constituents by UPLC ESI-MS/MS** | | | | | | |
| --- | --- | --- | --- | --- | --- | --- | --- |
| **No.** | | **Retention time (min.)** | **Identified metabolites** | **Molecular Formula** | **Precursor**  **Ions** | **MS/MS**  **product ions** | **Reference** |
|  | | 0.75 | Malic acid | C_4_H_6_O_5_ | [M-H]^-^ 133.0827 | 115.00 | (Taamalli *et al.*, 2015) |
|  | | 0.79 | Citric acid | C₆H₈O₇ | [M-H]^-^ 191.1 | 111.00 | (Taamalli *et al.*, 2015) |
|  | | 0.95 | Gluconic acid | C_6_H_12_O_7_ | [M-H]^-^ 195.1 | 129.01 | (Taamalli *et al.*, 2015) |
|  | | 1.42 | Fumaric acid | C_4_H_4_O_4_ | [M-H]^-^ 115.0627 | 115.00, 71.01 | (Taamalli *et al.*, 2015) |
|  | | 1.98 | Hydroquinone | C_6_H_6_O_2_ | [M+H]^+^ 111.1173 | 110, 11 | (Erenler *et al.*, 2016) |
|  | | 2.08 | Vanillic acid | C_8_H_8_O_4_ | [M+H]^+^ 169.1573 | 167, 152, 108 | (Taamalli *et al.*, 2015) |
|  | | 2.39 | Chlorogenic acid | C_16_H_18_O_9_ | [M-H]^-^ 353.1 | 173.47, 135.04, 191.05, 179.03 | (Taamalli *et al.*, 2015)  (Vallverdú-Queralt *et al.*, 2015) |
|  | | 2.43 | Gentisic acid | C_7_H_6_O_4_ | [M-H]^-^ 153.1127 | 152.9, 108.88 | (Erenler *et al.*, 2016) |
|  | | 3.7 | Sacranoside A | C_20_H_32_O_10_ | [M-H]^-^ 431.2 | 153.09, 385.18, 223.13 | (Taamalli *et al.*, 2015) |
|  | | 3.97 | Caffeic acid glucoside | C_15_H_18_O_9_ | [M-H]^-^ 341.2927 | 179, 161, 135 | (Hossain *et al.*, 2014) |
|  | | 5.18 | Luteolin rutinoside | C_27_H_30_O_16_ | [M-H]^-^ 593.2 | 285.00 | (Taamalli *et al.*, 2015)  (Hossain *et al.*, 2014) |
|  | | 5.68 | Caffeic acid | C_9_H_8_O_4_ | [M-H]^-^ 179.1527 | 135.00 | (Vallverdú-Queralt *et al.*, 2015) |
|  | | 5.81 | Ferulic acid | C_10_H_10_O_4_ | [M-H]^-^ 193.1727 | 193.00 , 178.00, 149.00, 134.00 | (Vallverdú-Queralt *et al.*, 2015) |
|  | | 6.08 | Thymohydroquinone | C_10_H_14_O_2_ | [M-H]^-^ 165.2127 | 151.00, 105.00, 39.00, 166.00, 95.00 | (Bouyahya & Jamal, 2016) |
|  | | 6.25 | procumboside B | C_15_H_22_O_10_ | [M-H]^-^ 361.33 | 361.16 | (Wang *et al.*, 2021) |
|  | | 6.72 | Carvacrol 2-O-β-glucopyranosyl-(1-2)-β-glucopyranosid | C_22_H_34_O_11_ | [M-H]^-^ 473.3 | 473.30 | (Kamel *et al.*, 2001) |
|  | | 6.78 | Rutin | C_27_H_30_O_16_ | [M-H]^-^ 609.2 | 611.15 | (Taamalli *et al.*, 2015) |
|  | | 6.85 | Caffeoyl-arbutin | C_21_H_22_O_10_ | [M-H]^-^ 433.1 | 323.07, 161.02 | (Taamalli *et al.*, 2015) |
|  | | 6.95 | Kaempferol-O-sambubioside | [C_26_H_28_O_15_](https://pubchem.ncbi.nlm.nih.gov/#query=C26H28O15) | [M-H]^-^ 579.2 | 285.04 | (Taamalli *et al.*, 2015) |
|  | | 7.15 | Luteolin glucuronide | C_21_H_18_O_12_ | [M-H]^-^ 461.1 | 285.00 | (Taamalli *et al.*, 2015) |
|  | | 7.25 | salvianolic acid C | C_26_H_20_O_10_ | [M-H]^-^ 537.4527 | 491.09, 311.05, 179.03 | (Lin *et al.*, 2003) |
|  | | 7.29 | diosmetin-7-glucuronide | C_22_H_20_O_12_ | [M-H]^-^ 475.3927 | 475.08, 299.05 | (Goel & Vasudeva, 2018) |
|  | | 7.55 | 2-Isopropyl-5-methylphenol (Thymol) | C_10_H_14_O | [M-H]^-^ 149.2127 | 149.09, 151.11, 97.05, 93.06, 81.06, 43.05 | (Raina & Negi, 2012) |
|  | | 7.59 | Hesperidin | C_28_H_34_O_15_ | [M-H]^-^ 609.5927 | 301.07 | (Taamalli *et al.*, 2015) |
|  | | 7.65 | Apigenin-O-glucuronide | [C_21_H_18_O_11_](https://pubchem.ncbi.nlm.nih.gov/#query=C21H18O11) | [M-H]^-^ 445.1 | 269.04, 175.02, 113.02 | (Taamalli *et al.*, 2015) |
|  | | 7.69 | Thymoquinone | C_10_H_12_O_2_ | [M-H]^-^ 163.1 | 163.05 | (Abdelaali *et al.*, 2021) |
|  | | 7.82 | Rosmarinic acid | C_18_H_16_O_8_ | [M-H]^-^ 359.1 | 179.00, 161.00, 135.00 | (Taamalli *et al.*, 2015)  (Erenler *et al.*, 2016) |
|  | | 7.99 | Quercitrin | C_21_H_20_O_11_ | [M-H]^-^ 447.3927 | 445.07 | (Taamalli *et al.*, 2015)  (Erenler *et al.*, 2016) |
|  | | 8.06 | Didymin | C_28_H_34_O_14_ | [M-H]^-^ 593.2 | 593.18, 285.07 | (Goel & Vasudeva, 2018) |
|  | | 8.09 | Tilianin | C_22_H_22_O_10_ | [M-H]^-^ 445.3927 | 284.00 | (Goel & Vasudeva, 2018) |
|  | | 8.69 | Gastrodin | C_13_H_18_O_7_ | [M-H]^-^ 285.1 | 123.04, 285.09, 105.03 | (H. Liu *et al.*, 2012) |
|  | | 9.53 | Kaempferol-3-O-glucoside (Astragaline) | C_21_H_20_O_11_ | [M-H]^-^ 447.3927 | 285.00 | (Vallverdú-Queralt *et al.*, 2015) |
|  | | 9.03 | Luteolin-7-O-glucoside (Cynaroside) | C_21_H_19_O_11_ | [M-H]^-^ 447.3927 | 285.00 | (Hossain *et al.*, 2014) |
|  | | 9.43 | (+)-lariciresinol | C_20_H_24_O_6_ | [M-H]^-^ 359.1 | 359.14 | (Wang *et al.*, 2021) |
|  | | 9.43 | Coumaric acid | C_9_H_8_O_3_ | [M-H]^-^ 163.1527 | 119.00 | (Vallverdú-Queralt *et al.*, 2015) |
|  | | 9.6 | Arbutin | C_12_H_16_O_7_ | [M-H]^-^ 271.05 | 253.00, 108.00 | (Taamalli *et al.*, 2015) |
|  | | 9.83 | Orientin | C_21_H_20_O_11_ | [M-H]^-^ 447.3927 | 357.05, 327.05 | (Taamalli *et al.*, 2015) |
|  | | 10.1 | Epigallocatechin | C_15_H_14_O_7_ | [M-H]^-^ 305.2627 | 289.00, 225.00 | (Hossain *et al.*, 2014) |
|  | | 10.3 | Quinic acid | C_7_H_12_O_6_ | [M-H]^-^ 191.1 | 127.03 | (Taamalli *et al.*, 2015) |
|  | | 10.47 | Origanine A | C_29_H_30_O_16_ | [M-H]^-^ 633.4927 | 589.15, 633.14 | (H. Liu *et al.*, 2012) |
|  | | 10.75 | Hesperetin | C_16_H_14_O_6_ | [M+H]^+^ 303.1 | 301.07, 287.05 | (Erenler *et al.*, 2016) |
|  | | 11 | Gallocatechin | C_15_H_14_O_7_ | [M-H]^-^ 305.2627 | 225.11 | (Taamalli *et al.*, 2015) |
|  | | 11.41 | Origanine B/C | C_38_H_38_O_20_ | [M-H]^-^ 813.6927 | 813.18, 571.14 | (Taamalli *et al.*, 2015) |
|  | | 11.64 | Lithospermic acid | C_27_H_22_O_12_ | [M-H]^-^ 537.2 | 159.00, 203.00, 223.00, 253.00, 267.00, 295.00, 313.00, 383.00, 537.00 | (G. V. Rao *et al.*, 2011) |
|  | | 12.4 | Carnosic acid | C_20_H_28_O_4_ | [M+H]^+^ 333.2 | 333.20, 315.19, 287.20 | (Bina & Rahimi, 2017) |
|  | | 12.71 | Caftaric acid | C_13_H_12_O_9_ | [M-H]^-^ 311.2 | 149.00 | (Vallverdú-Queralt *et al.*, 2015) |
|  | | 13.7 | Quercetin arabinoside | C_20_H_18_O_11_ | [M-H]^-^ 433.2927 | 301.00 | (Hossain *et al.*, 2014) |
|  | | 14.07 | Carnosol | C_20_H_26_O_4_ | [M+H]^+^ 331.4073 | 331.19 | (Bina & Rahimi, 2017) |
|  | | 14.92 | Narigenin-O-hexoside | C_21_H_22_O_10_ | [M-H]^-^ 433.3927 | 271.00 | (Vallverdú-Queralt *et al.*, 2015) |
|  | | 15.16 | Salvianolic acid I | C_27_H_22_O_12_ | [M-H]^-^ 537.4527 | 493.11, 339.05, 295.06, 197.04,135.04 | (Taamalli *et al.*, 2015) |
|  | | 15.49 | Globoidnan A | C_26_H_20_O_10_ | [M-H]^-^ 491.3927 | 311.05, 267.06, 197.85 | (Erenler *et al.*, 2017) |
|  | | 17.91 | Triacontanol | C_30_H_62_O | [M-H]^-^ 437.7927 | 437.47 | (G. V. Rao *et al.*, 2011) |
|  | | 18.86 | Taxifolin | C_15_H_12_O_7_ | [M+H]^+^ 305.3 | 285.04, 125.02 | (Taamalli *et al.*, 2015) |
|  | | 20.35 | Quercetin | C_15_H_10_O_7_ | [M-H]^-^ 301.2227 | 285.00, 227.10, 151.10, 135.00 | (Hossain *et al.*, 2014) |
|  | | 20.55 | Kaempferol | C_15_H_10_O_6_ | [M-H]^-^ 285.2327 | 285.00, 151.00 | (Vallverdú-Queralt *et al.*, 2015) |
|  | | 20.79 | Naringenin | C_15_H_12_O_5_ | [M-H]^-^ 271.2 | 271.00, 151.00 | (Vallverdú-Queralt *et al.*, 2015) |
|  | | 20.88 | Ursolic acid | C_30_H_48_O_3_ | [M-H]^-^ 455.6927 | 456.35 | (G. V. Rao *et al.*, 2011) |
|  | | 21.16 | Morin | C_15_H_10_O_7_ | [M-H]^-^ 301.2227 | 300.02, 299.09, 317.03 | (Erenler *et al.*, 2016) |
|  | | 21.35 | oleanolic acid | C_30_H_48_O_3_ | [M-H]^-^ 455.4 | 455.34, 54.00 | (G. V. Rao *et al.*, 2011) |
|  | | 21.57 | Apigenin | C_15_H_10_O_5_ | [M+H]^+^ 271.3 | 271.24, 151.00, 225.05, 119.04 | (Hossain *et al.*, 2014) |
|  | | 21.69 | Luteolin | C_15_H_10_O_6_ | [M-H]^-^ 285.1 | 151.00, 133.03 | (Taamalli *et al.*, 2015) |
|  | | 21.75 | Linolenic | C_18_H_30_O_2_ | [M-H]^-^ 277.3927 | 279.00, 54.00 | (Goel & Vasudeva, 2018) |
|  | | 21.78 | Dihydroquercetin | C_15_H_12_O_7_ | [M+H]^+^ 305.2573 | 301.00, 285.00, 241.00, 227.00, 151.00, 135.00 | (Hossain *et al.*, 2014) |
|  | | 22.39 | Amentoflavone | C_30_H_18_O_10_ | [M-H]^-^ 537.4927 | 537.08 | (Goel & Vasudeva, 2018) |
|  | | 22.46 | Isorhamnetin (O-Methyl-quercetin) | C_16_H_12_O_7_ | [M-H]^-^ 315.2527 | 300.02 | (Taamalli *et al.*, 2015) |
|  | | 22.68 | Linoleic acid | C_18_H_32_O_2_ | [M+H]^+^ 281.2 | 280.00, 281.00, 202.00, 203.00, 124.00, 54.00 | (Goel & Vasudeva, 2018) |
|  | | 22.73 | Hydroperoxy octadecadienoic acid | C_18_H_32_O_4_ | [M-H]^-^ 311.2 | 223.17, 54.00 | (Taamalli *et al.*, 2015) |
|  | | 23.72 | Sebacic acid | C_10_H_18_O_4_ | [M+H]^+^ 203.3 | 201.04, 183.10, 113.09 | (Taamalli *et al.*, 2015) |
|  | | 25.51 | 3-O-methyl-catechin (meciadanol) | C_16_H_16_O_6_ | [M-H]^-^ 303.2827 | 96.95 | (Taamalli *et al.*, 2015) |
|  | | 26.03 | Oleic acid | C_18_H_34_O_2_ | [M+H]^+^ 283.3 | 283.00, 263.00, 237.00, 54.00 | (Goel & Vasudeva, 2018) |

**Table S2. Potential protein targets of marjoram:**

| **Short name of protein** | **Full name of protein** | **Uniport ID** | **Interacting compound (s) (combined interaction score)** |
| --- | --- | --- | --- |
| SHBG | Sex Hormone Binding Globulin | P04278 | lariciresinol sulphate (0.53), 2-(4-hydroxy-3-methoxyphenyl)-4H-chromene-3,4,5,7-tetraol (0.33) |
| PPARG | Peroxisome Proliferator Activated Receptor Gamma | P37231 | Glycine linoleamide (0.73), Oleoylglycine (0.7) , 16-hydroxyoctadec-9-enoic acid (0.62) , oleic (1) |
| CYP19A1 | Cytochrome P450 Family 19 Subfamily A Member 1 | P11511 | Apigenin (1) |
| AR | Androgen Receptor | P10275 | Apigenin (1) |
| VEGFA | Vascular Endothelial Growth Factor A | P15692 | Sebacic acid glucuronide (0.3), Gastrodin (0.43) , catechin (0.56) , Apigenin (0.69) |
| ESR1 | Estrogen Receptor 1 | P03372 | Dihydroisorhamnetin (0.42), Apigenin (1) |
| CYP1A1 | Cytochrome P450 Family 1 Subfamily A Member 1 | P04798 | Apigenin (0.48) |
| ESR2 | Estrogen Receptor 2 | Q92731 | Dihydroisorhamnetin (0.42), Apigenin (1) |
| AKR1C3 | Aldo-Keto Reductase Family 1 Member C3 | P42330 | Caffeic acid 4-O-sulphate (0.41), Caftaric acid (0.52) |
| GSK3B | Glycogen Synthase Kinase 3 Beta | P49841 | Apigenin (1) |
| PPARD | Peroxisome Proliferator Activated Receptor Delta | Q03181 | Oleic (1) |
| MTNR1B | Melatonin Receptor 1B | P49286 | Vanilloylglycine (0.45) |
| CYP2C9 | Cytochrome P450 Family 2 Subfamily C Member 9 | P11712 | Vanilloylglycine (0.41) |
| MMP9 | Matrix Metallopeptidase 9 | P14780 | Caffeic cid L-carnitine (0.49), Caffeic acid 4-O-sulphate (0.6), Caftaric acid (0.58) |
| HSD17B2 | Hydroxysteroid 17-Beta Dehydrogenase 2 | P37059 | Vanilloylglycine (0.54), Apigenin (0.48) |
| MMP2 | Matrix Metallopeptidase 2 | P08253 | Caffeic acid 4-O-sulphate (0.6), Caftaric acid (0.58) |
| HMGCR | 3-Hydroxy-3-Methylglutaryl-CoA Reductase | P04035 | Glycine linoleamide (0.45), Oleoylglycine (0.49), Sebacic acid (0.52) , 16-hydroxyoctadec-9-enoic acid (0.44) , oleic (0.65) |
| PPARA | Peroxisome Proliferator Activated Receptor Alpha | Q07869 | Glycine linoleamide (0.73), oleic (1) |
| MTNR1A | Melatonin Receptor 1A | P48039 | Vanilloylglycine (0.45) |
| HSD17B1 | Hydroxysteroid 17-Beta Dehydrogenase 1 | P14061 | Apigenin (1) |
| GSTM1 | Glutathione S-Transferase Mu 1 | P09488 | Oleoylglycine (0.4) |
| DPP4 | Dipeptidyl Peptidase 4 | P27487 | Apigenin (1) |
| CNR1 | Cannabinoid Receptor 1 | P21554 | Linolenic acid L-carnitine (0.49), Glycine linoleamide (0.7) , Oleoylglycine (0.62) , 16-hydroxyoctadec-9-enoic acid (0.44), oleic (0.7) |
| MMP1 | Matrix Metallopeptidase 1 | P03956 | Caffeic cid L-carnitine (0.49) , Caffeic acid 4-O-sulphate (0.6), Caftaric acid (0.6) |
| FAAH | Fatty Acid Amide Hydrolase | O00519 | Linolenic acid L-carnitine (0.49), Glycine linoleamide (0.91) , Oleoylglycine (0.81) , 16-hydroxyoctadec-9-enoic acid (0.44), oleic (0.63) |
| XDH | Xanthine Dehydrogenase | P47989 | Quercitrin-4'-glucuronide (0.46), Apigenin (1) |
| TLR2 | Toll Like Receptor 2 | O60603 | Linolenic acid L-carnitine (0.45) , Glycine linoleamide (0.45) , Oleoylglycine (0.5) , Sebacic acid (0.71) , 16-hydroxyoctadec-9-enoic acid (0.44) , oleic (0.74) |
| IL2 | Interleukin 2 | P60568 | Origanine L-glycine (0.44) , Gastrodin (0.46) , Origanine B/C (0.42) , Apigenin (0.44) |
| CYP1B1 | Cytochrome P450 Family 1 Subfamily B Member 1 | Q16678 | Caffeic acid 4-O-sulphate (0.4), Quercitrin-4'-glucuronide (0.5), catechin (0.41) , 2-(4-hydroxy-3-methoxyphenyl)-4H-chromene-3,4,5,7-tetraol (0.41) , Dihydroisorhamnetin (0.62), Apigenin (1) |
| MPO | Myeloperoxidase | P05164 | Vanilloylglycine (0.4), Apigenin (1) |
| TTR | Transthyretin | P02766 | Vanilloylglycine (0.4), Caffeic cid L-carnitine (0.49), Caffeic acid 4-O-sulphate (0.41), Caftaric acid (0.6), Apigenin (1) |
| TRPV1 | Transient Receptor Potential Cation Channel Subfamily V Member 1 | Q8NER1 | Vanilloylglycine (0.45), Glycine linoleamide (0.63), Oleoylglycine (0.55) |
| AHR | Aryl Hydrocarbon Receptor | P35869 | Apigenin (0.47) |
| FGF2 | Fibroblast Growth Factor 2 | P09038 | Malic acid glucuronide (0.41), Thymol-3-glucouronide (0.38), Sebacic acid glucuronide (0.41), Gastrodin (0.43) |
| SELP | Selectin P | P16109 | Sebacic acid glucuronide (0.3), Glycine linoleamide (0.4), Oleoylglycine (0.43) |
| NDUFA6 | NADH:Ubiquinone Oxidoreductase Subunit A6 | P56556 | lariciresinol sulphate (0.7), 7-hydroxyLariciresinol (Tanegool) (0.49) |
| NDUFS4 | NADH:Ubiquinone Oxidoreductase Subunit S4 | O43181 | lariciresinol sulphate (0.7) , 7-hydroxyLariciresinol (Tanegool) (0.49) |
| SLC22A3 | Solute Carrier Family 22 Member 3 | O75751 | 7-hydroxyLariciresinol (Tanegool) (0.4) |
| GABRB1 | Gamma-Aminobutyric Acid Type A Receptor Subunit Beta1 | P18505 | Thymohydroquinone (0.39) |
| NDUFA5 | NADH:Ubiquinone Oxidoreductase Subunit A5 | Q16718 | lariciresinol sulphate (0.7), 7-hydroxyLariciresinol (Tanegool) (0.49) |
| NEK6 | NIMA Related Kinase 6 | Q9HC98 | Apigenin (0.48) |
| CYP3A4 | Cytochrome P450 Family 3 Subfamily A Member 4 | P08684 | Vanilloylglycine (0.41), Apigenin (1) |
| CXCL12 | C-X-C Motif Chemokine Ligand 12 | P48061 | Vanilloylglycine (0.44), Caftaric acid (0.46) , 2-(4-hydroxy-3-methoxyphenyl)-4H-chromene-3,4,5,7-tetraol (0.31) |
| CBS | Cystathionine Beta-Synthase | P35520 | Quercitrin-4'-glucuronide (0.53) , Dihydroisorhamnetin (0.57), Apigenin (0.55) |
| ALB | Albumin | P02768 | Thymol-3-glucouronide (0.31) |
| NFKB1 | Nuclear Factor Kappa B Subunit 1 | P19838 | Vanilloylglycine (0.49), Caffeic acid 4-*O*-sulphate (0.4) |
| GABBR2 | Gamma-Aminobutyric Acid Type B Receptor Subunit 2 | O75899 | Sebacic acid (0.59) |
| MAOA | Monoamine Oxidase A | P21397 | Apigenin (1) |
| ROCK1 | Rho Associated Coiled-Coil Containing Protein Kinase 1 | Q13464 | Vanilloylglycine (0.43) |
| CYP1A2 | Cytochrome P450 Family 1 Subfamily A Member 2 | P05177 | Vanilloylglycine (0.4), Apigenin (1) |
| ALDH1B1 | Aldehyde Dehydrogenase 1 Family Member B1 | P30837 | Origanine L-glycine (0.36), Gastrodin (0.46), Origanine B/C (0.35) |
| HRAS | HRas Proto-Oncogene, GTPase | P01112 | Origanine L-glycine (0.35), Origanine B/C (0.35) |
| CFTR | CF Transmembrane Conductance Regulator | P13569 | Apigenin (1) |
| CREB1 | CAMP Responsive Element Binding Protein 1 | P16220 | Apigenin (0.47) |
| ACHE | Acetylcholinesterase (Yt Blood Group) | P22303 | Dihydroisorhamnetin (0.48), Apigenin (0.45) |
| PGF | Placental Growth Factor | P49763 | Apigenin (0.69) |

**Table S3. Marjoram main active component network node topological parameters**

| **Compound name** | **Betweenness Centrality** | **Closeness Centrality** | **Degree** |
| --- | --- | --- | --- |
| Apigenin | 0.54674428 | 0.35159817 | 25 |
| Vanilloylglycine | 0.37809931 | 0.31950207 | 12 |
| Glycine linoleamide | 0.09687317 | 0.24758842 | 8 |
| Oleic | 0.08849875 | 0.23404255 | 8 |
| Oleoylglycine | 0.107482 | 0.24758842 | 8 |
| Caffeic acid 4-O-sulphate | 0.07922142 | 0.28 | 8 |
| Caftaric acid | 0.03608799 | 0.27017544 | 6 |
| 7-hydroxyLariciresinol | 0.02648667 | 0.14076782 | 4 |
| 16-hydroxyoctadec-9-enoic acid | 9.63E-04 | 0.17460317 | 4 |
| Dihydroisorhamnetin | 0.00590907 | 0.23404255 | 4 |
| Gastrodin | 0.0743655 | 0.24919094 | 4 |
| lariciresinol sulphate | 0.12354751 | 0.19012346 | 4 |
| Caffeic cid L-carnitine | 0.00761007 | 0.25081433 | 3 |
| Origanine L-glycine | 0.01698102 | 0.22063037 | 3 |
| 2-(4-hydroxy-3-methoxyphenyl)-4H-chromene-3,4,5,7-tetraol | 0.17242967 | 0.28 | 3 |
| Quercitrin-4'-glucuronide | 0.00377324 | 0.2326284 | 3 |
| Sebacic acid glucuronide | 0.09952789 | 0.27017544 | 3 |
| Sebacic acid | 0.02608035 | 0.1738149 | 3 |
| Origanine B/C | 0.01698102 | 0.22063037 | 3 |
| Linolenic acid L-carnitine | 1.44E-04 | 0.17303371 | 2 |
| catechin | 0.01461381 | 0.26460481 | 2 |
| Thymol-3-glucouronide | 0.02597403 | 0.18465228 | 2 |
| Thymohydroquinone | 0 | 1 | 1 |
| isovaleryl glucuronide | 0 | 0.18377088 | 1 |

**Table S4. Marjoram main active ingredient target network node topological parameters**

| **Gene name** | **Betweenness Centrality** | **Closeness Centrality** | **Degree** | **Gene name** | **Betweenness Centrality** | **Closeness Centrality** | **Degree** |
| --- | --- | --- | --- | --- | --- | --- | --- |
| CYP1B1 | 0.15583391 | 0.2972973 | 6 | CYP1A2 | 0.03642594 | 0.30923695 | 2 |
| TLR2 | 0.0455676 | 0.20754717 | 6 | SHBG | 0.14559125 | 0.22713864 | 2 |
| TTR | 0.12641181 | 0.32765957 | 5 | MPO | 0.03642594 | 0.30923695 | 2 |
| HMGCR | 0.03231478 | 0.20643432 | 5 | PPARA | 5.45E-04 | 0.20104439 | 2 |
| FAAH | 0.01985939 | 0.20533333 | 5 | MMP2 | 4.13E-04 | 0.22190202 | 2 |
| IL2 | 0.1080097 | 0.27797834 | 4 | GABRB1 | 0 | 1 | 1 |
| FGF2 | 0.08592254 | 0.2244898 | 4 | ACHE | 0 | 0.26101695 | 1 |
| TRPV1 | 0.25828585 | 0.28 | 4 | HSD17B1 | 0 | 0.26101695 | 1 |
| PPARG | 0.00740401 | 0.20424403 | 4 | PGF | 0 | 0.26101695 | 1 |
| VEGFA | 0.12154274 | 0.30677291 | 4 | CREB1 | 0 | 0.26101695 | 1 |
| ALDH1B1 | 0.00632376 | 0.20316623 | 3 | CYP1A1 | 0 | 0.26101695 | 1 |
| CNR1 | 0.00142315 | 0.20316623 | 3 | ROCK1 | 0 | 0.24290221 | 1 |
| MMP9 | 0.00208794 | 0.22318841 | 3 | GSK3B | 0 | 0.26101695 | 1 |
| SELP | 0.06768612 | 0.24444444 | 3 | CYP19A1 | 0 | 0.26101695 | 1 |
| MMP1 | 0.00208794 | 0.22318841 | 3 | ALB | 0 | 0.15618661 | 1 |
| ESR1 | 0.02007579 | 0.27402135 | 3 | CYP2C9 | 0 | 0.24290221 | 1 |
| CXCL12 | 0.08982345 | 0.27208481 | 3 | CFTR | 0 | 0.26101695 | 1 |
| CBS | 0.01011389 | 0.26460481 | 3 | MTNR1B | 0 | 0.24290221 | 1 |
| ESR2 | 0.00414035 | 0.26279863 | 2 | AHR | 0 | 0.26101695 | 1 |
| NDUFA5 | 0.01663249 | 0.16142558 | 2 | PPARD | 0 | 0.19012346 | 1 |
| HSD17B2 | 0.03642594 | 0.30923695 | 2 | MAOA | 0 | 0.26101695 | 1 |
| HRAS | 1.14E-04 | 0.1820331 | 2 | SLC22A3 | 0 | 0.12359551 | 1 |
| NFKB1 | 0.01412789 | 0.25925926 | 2 | GSTM1 | 0 | 0.19896641 | 1 |
| NDUFA6 | 0.01663249 | 0.16142558 | 2 | NEK6 | 0 | 0.26101695 | 1 |
| AKR1C3 | 4.13E-04 | 0.22190202 | 2 | MTNR1A | 0 | 0.24290221 | 1 |
| NDUFS4 | 0.01663249 | 0.16142558 | 2 | GABBR2 | 0 | 0.14836224 | 1 |
| XDH | 0.00562038 | 0.26279863 | 2 | DPP4 | 0 | 0.26101695 | 1 |
| CYP3A4 | 0.03642594 | 0.30923695 | 2 | AR | 0 | 0.26101695 | 1 |

**Table S5. KEGG pathway analysis of potential target gene functions:**

| **#term ID** | **term description** | **observed gene count** | **false discovery rate (p value)** | **matching proteins in your network (labels)** |
| --- | --- | --- | --- | --- |
| hsa00140 | Steroid hormone biosynthesis | 8 | 2.70E-09 | HSD17B2, HSD17B1, CYP1A2, AKR1C3, CYP1A1, CYP19A1, CYP1B1, CYP3A4 |
| hsa04915 | Estrogen signaling pathway | 7 | 7.85E-06 | MMP2, GABBR2, ESR2, MMP9, CREB1, ESR1, HRAS |
| hsa04913 | Ovarian steroidogenesis | 6 | 1.28E-06 | HSD17B2, HSD17B1, AKR1C3, CYP1A1, CYP19A1, CYP1B1 |
| hsa04723 | Retrograde endocannabinoid signaling | 6 | 9.14E-05 | FAAH, GABRB1, NDUFS4, CNR1, NDUFA5, NDUFA6 |
| hsa04024 | cAMP signaling pathway | 6 | 0.00051 | CFTR,NFKB1,GABBR2,ROCK1,PPARA,CREB1 |
| hsa00380 | Tryptophan metabolism | 5 | 9.18E-06 | MAOA, CYP1A2, ALDH1B1, CYP1A1, CYP1B1 |
| hsa04917 | Prolactin signaling pathway | 5 | 5.49E-05 | NFKB1, GSK3B, ESR2, ESR1, HRAS |
| hsa01522 | Endocrine resistance | 5 | 0.00016 | MMP2, ESR2, MMP9, ESR1, HRAS |
| hsa05224 | Breast cancer | 5 | 0.00095 | FGF2, GSK3B, ESR2, ESR1, HRAS |
| hsa04062 | Chemokine signaling pathway | 5 | 0.0021 | NFKB1, GSK3B, CXCL12, ROCK1, HRAS |
| hsa03320 | PPAR signaling pathway | 4 | 0.00096 | PPARG, PPARD, MMP1, PPARA |
| hsa04211 | Longevity regulating pathway | 4 | 0.0014 | NFKB1, PPARG, CREB1, HRAS |
| hsa04657 | IL-17 signaling pathway | 4 | 0.0016 | NFKB1, MMP1, GSK3B, MMP9 |
| hsa04660 | T cell receptor signaling pathway | 4 | 0.0021 | NFKB1, IL2, GSK3B, HRAS |
| hsa04931 | Insulin resistance | 4 | 0.0025 | NFKB1, GSK3B, PPARA, CREB1 |
| hsa04152 | AMPK signaling pathway | 4 | 0.0035 | CFTR, PPARG, HMGCR, CREB1 |
| hsa04015 | Rap1 signaling pathway | 4 | 0.0147 | FGF2, CNR1, HRAS, PGF |
| hsa04014 | Ras signaling pathway | 4 | 0.0191 | NFKB1, FGF2, HRAS, PGF |
| hsa04010 | MAPK signaling pathway | 4 | 0.0347 | NFKB1, FGF2, HRAS, PGF |
| hsa00591 | Linoleic acid metabolism | 3 | 0.0012 | CYP2C9, CYP1A2, CYP3A4 |
| hsa04929 | GnRH secretion | 3 | 0.0062 | GABBR2, ESR2, HRAS |
| hsa04662 | B cell receptor signaling pathway | 3 | 0.01 | NFKB1, GSK3B, HRAS |
| hsa04933 | AGE-RAGE signaling pathway in diabetic complications | 3 | 0.0147 | MMP2, NFKB1, HRAS |
| hsa04625 | C-type lectin receptor signaling pathway | 3 | 0.0156 | NFKB1, IL2, HRAS |
| hsa04668 | TNF signaling pathway | 3 | 0.0194 | NFKB1, MMP9, CREB1 |
| hsa04722 | Neurotrophin signaling pathway | 3 | 0.0196 | NFKB1, GSK3B, HRAS |
| hsa04071 | Sphingolipid signaling pathway | 3 | 0.0213 | NFKB1, ROCK1, HRAS |
| hsa00190 | Oxidative phosphorylation | 3 | 0.0251 | NDUFS4, NDUFA5, NDUFA6 |
| hsa04550 | Signaling pathways regulating pluripotency of stem cells | 3 | 0.0317 | FGF2, GSK3B, HRAS |
| hsa00340 | Histidine metabolism | 2 | 0.0113 | MAOA, ALDH1B1 |
| hsa00260 | Glycine, serine and threonine metabolism | 2 | 0.0241 | MAOA, CBS |
| hsa00330 | Arginine and proline metabolism | 2 | 0.0333 | MAOA, ALDH1B1 |
| hsa05213 | Endometrial cancer | 2 | 0.0449 | GSK3B, HRAS |
| hsa00590 | Arachidonic acid metabolism | 2 | 0.0473 | CYP2C9, AKR1C3 |
